# Epigenetic Targeting to Overcome Radioresistance in Head and Neck Cancer

**DOI:** 10.3390/cancers16040730

**Published:** 2024-02-09

**Authors:** Iñaki Schniewind, Maria José Besso, Sebastian Klicker, Franziska Maria Schwarz, Wahyu Wijaya Hadiwikarta, Susan Richter, Steffen Löck, Annett Linge, Mechthild Krause, Anna Dubrovska, Michael Baumann, Ina Kurth, Claudia Peitzsch

**Affiliations:** 1 National Center for Tumor Diseases (NCT), Faculty of Medicine, University Hospital Carl Gustav Carus, Dresden University of Technology, 01307 Dresden, Germany; inaki.schniewind@ukdd.de (I.S.);; 2 OncoRay—National Center for Radiation Research in Oncology, Faculty of Medicine, University Hospital Carl Gustav Carus, Dresden University of Technology and Helmholtz-Zentrum Dresden-Rossendorf, 01307 Dresden, Germany; 3 Department of Neurology, Carl Gustav Carus University Hospital, Dresden University of Technology, 01307 Dresden, Germany; 4 German Cancer Research Center (DKFZ), Division Radiooncology/Radiobiology, 69120 Heidelberg, Germany; 5 German Cancer Consortium (DKTK), Partner Site Dresden, 01307 Dresden, Germany; 6 Institute of Radiooncology—OncoRay, Helmholtz-Zentrum Dresden-Rossendorf (HZDR), 01307 Dresden, Germany; 7 Institute of Clinical Chemistry and Laboratory Medicine, University Hospital Carl Gustav Carus, Dresden University of Technology, 01307 Dresden, Germany; 8 Department of Radiation Oncology, University Hospital Carl Gustav Carus, 01307 Dresden, Germany; 9 Center for Regenerative Therapies Dresden (CRTD), Dresden University of Technology, 01307 Dresden, Germany

**Keywords:** histone methylation, radioresistance, cellular plasticity, cancer stem cells, head and neck cancer

## Abstract

**Simple Summary:**

Cells with stem-like potential in head and neck squamous cell carcinoma (HNSCC) have been shown to exhibit features of intrinsic resistance to ionizing radiation. These cells, often referred to as cancer stem cells (CSCs), are characterized by an increased self-renewal and clonogenic potential, features associated with epithelial-to-mesenchymal transition (EMT), migratory behavior, and cellular plasticity. Cellular plasticity entails changes in phenotype and function in response to therapeutic pressures such as irradiation. Previously, we found that ALDH-positive CSCs are enriched in HNSCC cultures upon treatment with ionizing radiation. This process was characterized by mechanisms involving a selection of resistant populations and an induction of stem-like features. Cellular plasticity is regulated intracellularly through epigenetic mechanisms, including dynamic adaptations of DNA methylation and histone methylation, resulting in gene-specific alterations of expression levels. Therefore, we hypothesized that epigenetic targeting may prevent irradiation-induced cellular plasticity, rendering resistant HNSCC cells more sensitive. Employing a chemical library screen, we identified the histone demethylase inhibitor GSK-J1 to have a putative radiosensitizing effect that also reduces the stem-like potential of these cells.

**Abstract:**

(1) Background: The sensitivity of head and neck squamous cell carcinoma (HNSCC) to ionizing radiation, among others, is determined by the number of cells with high clonogenic potential and stem-like features. These cellular characteristics are dynamically regulated in response to treatment and may lead to an enrichment of radioresistant cells with a cancer stem cell (CSC) phenotype. Epigenetic mechanisms, particularly DNA and histone methylation, are key regulators of gene-specific transcription and cellular plasticity. Therefore, we hypothesized that specific epigenetic targeting may prevent irradiation-induced plasticity and may sensitize HNSCC cells to radiotherapy. (2) Methods: We compared the DNA methylome and intracellular concentrations of tricarboxylic acid cycle metabolites in radioresistant FaDu and Cal33 cell lines with their parental controls, as well as aldehyde dehydrogenase (ALDH)-positive CSCs with negative controls. Moreover, we conducted a screen of a chemical library targeting enzymes involved in epigenetic regulation in combination with irradiation and analyzed the clonogenic potential, sphere formation, and DNA repair capacity to identify compounds with both radiosensitizing and CSC-targeting potential. (3) Results: We identified the histone demethylase inhibitor GSK-J1, which targets UTX (*KDM6A*) and JMJD3 (*KDM6B*), leading to increased H3K27 trimethylation, heterochromatin formation, and gene silencing. The clonogenic survival assay after siRNA-mediated knock-down of both genes radiosensitized Cal33 and SAS cell lines. Moreover, high *KDM6A* expression in tissue sections of patients with HNSCC was associated with improved locoregional control after primary (*n* = 137) and post-operative (*n* = 187) radio/chemotherapy. Conversely, high *KDM6B* expression was a prognostic factor for reduced overall survival. (4) Conclusions: Within this study, we investigated cellular and molecular mechanisms underlying irradiation-induced cellular plasticity, a key inducer of radioresistance, with a focus on epigenetic alterations. We identified UTX (*KDM6A*) as a putative prognostic and therapeutic target for HNSCC patients treated with radiotherapy.

## 1. Introduction

Head and neck squamous cell carcinomas (HNSCC) are prevalent malignancies, accounting for about 900,000 new cases worldwide each year [1,2]. Many tumors are diagnosed in a locoregionally advanced stage, resulting in a 5-year survival rate of 40% to 60% despite multimodal treatment including surgery, radio- and chemotherapy [3,4]. Risk factors include tobacco smoking, alcohol consumption, and human papillomavirus (HPV) infection [5]. Although HPV infections increase the risk for oropharyngeal cancer, they correlate with a better prognosis than HPV-negative disease, e.g., after radio-chemotherapy.

Cancer stem cells (CSCs) play a crucial role in therapy resistance and relapse in HNSCC, owing to their capacity for self-renewal and differentiation [6,7]. This is evident in the use of CSC markers such as CD44, CD98, and c-MET as prognostic biomarkers in HNSCC patients treated with radiotherapy [8,9,10]. CSCs contribute to intra-tumor heterogeneity due to their high self-renewal and differentiation capacity and are associated with worse patient outcomes [11,12,13]. With cellular barcoding, it was shown that single cells with high CD98 expression and ALDH activity in HNSCC cell lines contribute to clonogenic survival after irradiation [14]. Moreover, it was demonstrated for various tumor entities that irradiation leads to an enrichment of CSCs with a radioresistant phenotype and elevated DNA repair capacity, controlled by metabolic and epigenetic mechanisms including DNA methylation, histone modification, and non-coding RNAs [6,15,16,17]. 

Notably, we demonstrated that the pharmacological inhibition of the histone methyltransferase (HMT) enhancer of zeste 2 polycomb repressive complex 2 (EZH2) using the chemical compound 3-deazaneplanocin A (DZNeP) resulted in the sensitization of radioresistant prostate cancer cells and reduced tumorigenicity [18,19]. Similarly, we identified epigenetic compounds that sensitized glioblastoma multiforme cell lines to photon and proton irradiation [20]. 

The Cancer Genome Atlas (TCGA) network revealed a high frequency of mutations and focal deletions in genes such as the histone 3 lysine 36 methyltransferase 2D (*KMTD2*, 5%) or the nuclear set domain gene (*NSD1/KMT3B*, 4%) in HNSCC [3]. Currently, there are several epigenetic targeting compounds under preclinical and clinical investigation for patients with HNSCC. Some of the compounds, such as the HDAC inhibitors vorinostat (SAHA) (phase II, NCT05608369), panobinostat (phase I, NCT00670553), and valproic acid (phase II, NCT01695122), are being tested in combination with radiotherapy (www.clinicaltrials.gov). 

Therefore, we hypothesized that epigenetic targeting might be a promising strategy to sensitize HNSCC cells to irradiation by affecting CSC plasticity and DNA repair. In this study, we conducted a chemical library screen of epigenetic targeting agents to identify CSC-targeting and radiosensitizing compounds in HNSCC cell lines. We identified the histone demethylase inhibitor GSK-J1, characterized the underlying molecular mechanisms, and validated the prognostic potential of the target proteins UTX (*KDM6A*) and JMJD3 (*KDM6B*).

## 2. Materials and Methods

### 2.1. Cell Culture

Within this study, the human head and neck squamous cell carcinoma (HNSCC) cell lines FaDu(DD) (RRID:CVCL_VP44, ATCC), SAS (RRID:CVCL_1675, Health Science Research Resources Bank, Osaka, Japan), Cal33 (RRID:CVCL_1108, Deutsche Sammlung von Mikroorganismen und Zellkulturen DSMZ GmbH), and UT-SCC-5 (RRID:CVCL_7858, University Turku, Finland) were used. Radioresistant (RR) sublines were generated from indicated parental cell lines via selection with at least 15 fractions of 4 Gy and analyzed together with age-matched controls [17]. All HNSCC cell lines were cultured in Dulbecco’s modified eagle medium (DMEM) supplemented with 10% fetal bovine serum (FBS), 1% L-Glutamine, 1% 2-(4-(2-Hydroxyethyl)-1-piperazinyl)-ethansulfonacid (HEPES), 1% minimum essential medium (MEM), 1% non-essential amino acid (NEAA), and 1% sodium pyruvate. The patient-derived xenograft (PDX) cell line DK19 and the immortalized human skin keratinocyte cell line HaCaT (RRID:CVCL_0038, DKFZ, Heidelberg, Germany) were used as controls and were cultured in DMEM supplemented with 1% NEAA, 1% HEPES, and 1% sodium pyruvate. All cell lines were used for experiments until passage 15, tested for mycoplasma infection and SNP-based cell authentication on a regular basis.

### 2.2. DNA Methylome Analysis

Genomic DNA was isolated in triplicate from each cell population with the Qiamp DNA Mini Kit (Qiagen, Hilden, Germany) according to the manufacturer’s recommendations. Approximately 1 microgram of DNA with a concentration of 25 ng/µL was used. Human DNA methylation bead array analysis with the EPIC array (v1_b4, Illumina, San Diego, CA, USA) was conducted at the Microarray Unit at the Genomics and Proteomics Core Facility (GPCF, DKFZ, Heidelberg, Germany). Raw data (IDAT format files) was imported and processed for differential methylation analysis with the R package RnBeads. Quality control was performed and data was normalized using the ‘wm.dasen’ method. Moreover, background normalization was performed using the ‘methylumi.noob’ method. The exploratory analysis module of RnBeads was activated to perform dimension reduction and statistical association tests to visualize and address association between methylation and experimental groups of interest. Beta values (β) estimating the methylation level, e.g., within gene bodies, promoter regions, and CpG islands, were calculated. Differential methylation analysis was conducted on site and at the regional level according to the sample groups specified in the analysis. *p*-values on the site level were computed using the limma method; i.e., hierarchical linear models from the limma package were employed and fitted using an empirical Bayes approach on derived M-values. Differential methylation was defined based on a 10% mean methylation difference between groups of interest and a false discovery rate (FDR)-adjusted *p*-value < 0.05 (Supplementary Table S2).

### 2.3. Mass Spectrometry-Based Analysis of Cellular Metabolisms

Seeded cells were treated, washed with ice-cold PBS, and harvested in 500 μL methanol. Extracts were centrifuged, and supernatants plus internal standard mix were dried in a speed vac concentrator. To determine the concentrations of Krebs cycle metabolites (succinate, fumarate, malate, citrate, isocitrate, cis-aconitate, α-ketoglutarate), 2-hydroxyglutarate, pyruvate, lactate, glutamate, glutamine, aspartate, and asparagine, liquid chromatography–tandem mass spectrometry (LC-MS/MS) was used as described previously at the experimental LC-MS/MS unit (Institute for Clinical Chemistry and Laboratory Medicine, Dresden, Germany). Briefly, on the day of analysis, dried samples were resuspended in 99/1 mobile phases A and B. A QTRAP 5500 triple quadrupole mass spectrometer (AB Sciex, Darmstadt, Germany) was coupled to an ACQUITY UPLC system from the Waters Corporation (Waters Corp., Eschborn, Germany) using an ACQUITY UPLC^®^ HSS T3 column (1.8 μm, 2.1 × 100 mm) with guard column (Waters Corp.) for separation. Mobile phases consisted of 0.2% formic acid in water (A) and 0.2% formic acid in acetonitrile. The MS was used in negative electrospray ionization and multiple-reaction monitoring scan mode. To quantify the metabolites, a comparison of the analyte peak areas to calibrator peak areas from the stable isotope labeled internal standards in samples was applied. Raw data were normalized to analyzed cell count and specified as pg per cell. 

### 2.4. Cell Irradiation

Cell irradiation was performed with the Maxishot Y.TU 320 machine (Yxlon International, Comet Group, Flamatt, The Switzerland) with 200 kV X-rays and a dose rate of 1.32 Gy/min at 20 mA. We used a collimator plate made of 7 mm beryllium and filtered the X-rays in the irradiation field (22 × 18 cm) with 0.5 mm copper. The absorbed dose was measured using a semiflex ionization chamber and the duplex dosimeter Unidos 11767 (PTW). Dose homogeneity was ensured through daily quality control and routine calibration. 

### 2.5. Chemical Library Screen of Epigenetic Targeting Agents

For the chemical library screen, FaDu and Cal33 cells were seeded in 96-well plates for colony formation, sphere formation, and γH2AX assay. The following day, the Epigenetics Screening Library (Cat#11076, Cayman Chemical, Ann Arbor, MI, USA) was added at a final concentration of 5 μM. The chemical library contains 146 small molecules that are known to modulate the activity of different epigenetic ‘writer’, ‘reader’, and ‘eraser’ proteins, such as DNA and histone methyltransferases, DNA and histone demethylases, histone acetyltransferases, histone deacetylases, and acetylated histone binding proteins. After an additional 24 h, the plates were irradiated with 4 Gy. Colonies were counted on day 10, spheres on day 14, and γH2AX foci were counted 24 h after irradiation. The screen was performed in biological duplicates, each replicate consisting of one well per compound and 46 DMSO control wells. Compounds exhibiting a clonogenic plating efficiency of less than 5% in the unirradiated control were considered cytotoxic and excluded from further analysis. For each assay and compound, the values of both cell lines were grouped and compared to the mean DMSO value using a one-tailed *t*-test. Compounds with a statistically significant lower clonogenic/spherogenic survival fraction or higher number of γH2AX foci compared to the DMSO control were identified as hits, i.e., as having a radiosensitizing effect in the respective assay. Selected hits from the screen were further validated using a 3D colony formation assay in combination with single dose irradiation. A concentration equal to the 5% inhibitory concentration (IC_5_) was used after establishing the dose-response relationship by assessing cell viability using the CellTiterGlo (Promega, Madison, WI, USA).

### 2.6. Histone Modification Assay

The nuclear extraction of cells grown in culture was performed with the EpiQuik™ Nuclear Extraction Kit I (#OP-0002) according to the manufacturer’s recommendations. Five to 10 µg nuclear extracts were analyzed with the EpiQuik Histone Demethylase (H3K4 Specific) Activity/Inhibition Assay Kit (#P-3074-48) and EpiQuik Histone Demethylase (H3K9 Specific) Activity/Inhibition Fast Assay Kit (#P-3077-48) to screen for histone demethylase activity in the different cells lines after irradiation as well as with the Epigenase™ JMJD2 Demethylase Activity/Inhibition Assay Kit (Fluorometric) (#P-3081-48), Epigenase JARID Demethylase Activity/Inhibition Assay Kit (Fluorometric) (#P-3083-48), and Epigenase JMJD3/UTX Demethylase Activity/Inhibition Assay Kit (Fluorometric) (#P-3085-48). The plates were scanned with the Tecan Infinite.

### 2.7. Cell Proliferation Assay

Cells were plated in 96-well plates with cell counts between 3000 to 8000 cells per well depending on the cell line; 24 h after plating, cells were treated with increasing inhibitor concentrations and cell survival after 24 h was analyzed using the Luminescent Cell Viability Assay (CellTiterGlo, Promega) according to the manufacturer’s recommendation. The luminescence signal was measured with the Tecan microplate reader, and the half maximal inhibitory concentration (IC_50_) was calculated with GraphPad Prism software (version 8).

### 2.8. 3D Colony Forming Assay

The clonogenic survival assay was performed on Matrigel (Fisher Scientific, Waltham, MA, USA)-based 3D cultures after 1 h pre-treatment with the defined 5% inhibitory concentration (IC_5_) of every compound with 2 Gy, 4 Gy, 6 Gy and 8 Gy irradiation. For this purpose, 1000 cells per well were seeded into 96-well culture plates coated with 50 µL of 1% low-melting agarose (Sigma Aldrich). A 100 µL quantity of cell suspension was mixed with Matrigel (1:20) and one hour before irradiation overlayed with 50 µL medium inhibitor containing media. We used the 5% inhibitory concentration (IC_5_) of GSK-J1 with 4.5 µM and the solvent dimethyl sulfoxide (DMSO) as control. The colonies were scanned with the Celigo S image Cytometer (Nexcelom Biosience, Lawrence, MA, USA) after 6 to 8 days and counted with a threshold of diameter > 50 µm. The plating efficiency and surviving fraction were calculated according to the following formulas:

$$Plating\;efficiency\left(PE\right)\left(\%\right)=\left(\frac{number\;of\;colonies}{number\;of\;plated\;cells}\right)\times 100$$

$$Surviving\;fraction(SF)=(\frac{PE\;at\;respective\;IR\;dose}{PE\;of\;sham\;control})$$

The obtained surviving fraction (SF) after a defined radiation dose (D) was fitted with a weighted, stratified, linear regression according to the linear–quadratic (LQ) formula: *SF*(*D*) = e^−αD−βD^2^ to determine the linear parameter α and the quadratic parameter β for different treatment conditions to perform group comparisons with the Statistical Package for the Social Sciences (SPSS) v23 software (IBM^®^, Chicago, IL, USA). To indicate the GSK-J1 inhibitor effect within the combined treatment together with irradiation, we calculated the dose modifying factor (DMF) as the ratio of doses with monotherapy versus a combination that causes the same level of biological effect. 

### 2.9. Sphere Formation Assay

To determine self-renewal properties, cells were grown under non-adherent conditions in growth factor-defined human mammary epithelial cell growth medium (MEBM) consisting of B27 (Sigma-Aldrich, St. Louis, MO, USA), epidermal growth factor (Peqlab, Erlangen, Germany; 20 ng/mL), fibroblast growth factor (20 ng/mL), insulin (4 µg/mL), 1% penicillin-streptomycin, and glutamine. Cells were added into 24-well ultra-low attachment plates, with 5000 cells in one milliliter per well. One hour before irradiation, the cells were treated with an IC_5_ concentration of GSK-J1 (4.5 µM) and cultured for 14 days. Spheres were scanned with the Celigo S image Cytometer (Nexcelom Bioscience, Lawrence, Massachusetts, USA) and counted with a diameter >100 µm.

### 2.10. Generation of Color-Coded Cell Lines

To generate HNSCC cell lines stably expressing blue fluorescent protein (BFP), tandem-dimer Tomato (tdTomato), or enhanced green fluorescent protein (eGFP), HEK293 cells were used for lentivirus production. The cells were transfected, 24 h after plating, with calcium phosphate containing 2 μg psPAX2, 1 μg pMD2.G, and 2.5 μg pWPXL plasmid (Addgene, Watertown, Massachusetts, USA). The original green fluorescent protein (GFP) insert was replaced with either mTagBFP (blue fluorescent protein) or tdTomato. The viruses were collected at 24, 48, and 96 h after transfection, pooled, and passed through a 45 μm filter (Sarstedt). Between 100,000 and 200,000 HNSCC cells per well were plated in a 6-well plate one day before transduction individually defined per cell line. To obtain pure BFP and tdTomato-expressing cell populations, cells with the highest 5% intensity were isolated with fluorescence-activated cell sorting (FACS, BD FACSAria™ III; BD Biosciences, Franklin Lakes, NJ, USA). The stable expression of the fluorescent proteins was examined via flow cytometry and revealed a purity of 98.6% for Cal33-BFP, 97.7% for Cal33-tdTomato, 51.4% for FaDu-BFP, and 96.8% for FaDu-tdTomato cells.

### 2.11. siRNA-Mediated Knockdown

To test the radiobiological effects of the genes *KDM6A* and *KDM6B* in HNSCC cells, 250,000 cells per well were plated in 6-well plates and treated 24 h later with 0.4 µL siRNA (40 pmol, Eurofins, Luxembourg City, Luxembourg) mixed with 9 µL of Lipofectamine RNAiMAX (Thermo Fisher, Waltham, MA, USA) in OptiMEM media (Thermo Fisher). Untreated, Lipofectamine-treated (MOCK control) and scrambled siRNA (Scrambled#3: ‘CCC UUC AAC UCG GGA GCA ATT’) transfected cells were used as controls. Three specific siRNA sequences to target *KDM6A* (KDM6A-2852: ‘GGU UUA CUA AGU UCA GAC AAT’, KDM6A-738: ‘GGU ACU ACA GUU UAC AGU CTG’, KDM6A-4523: ‘GCU CAU UAC UGUAGC AUU UGT’) and *KDM6B* (KDM6B-3120: ‘GGU GCU AGA ATA GAU CAG CCG’, KDM6B-3657: ‘GCU GCG CUC ACU UAF UFA GGG’, KDM6B-1715: ‘GCA GUC GGA AAC CGU UCU UGG’) were used for each transcript. Treated cells were seeded into 96-well plates with 1500 cells in each well, plated for clonogenic survival assay, and irradiated for 24 h after knock-down. The knock-down efficiency was validated on protein level via Western blot.

### 2.12. Western Blot

Cell cultures were washed with PBS and lysed with radioimmunoprecipitation assay (RIPA) buffer with cell scraper on ice. After centrifugation, protein extracts were measured with the bicinchoninic acid (BCA) assay. Bovine serum albumin (BSA) was used as reference to calculate the protein concentration. Equally protein concentrations between different samples were applied. Before loading the NuPAGE 1–12% gels (Invitrogen), samples were mixed with 4× SDS sample puffer including dithiothreitol (DDT) for 5 min at 95 °C. After protein separation according to their kDa size within the gel, they were transferred to an 0.2 µm nitrocellulose membrane (Amersham Protran, GE Healthcare, Chicago, IL, USA). The semi-dry transfer was performed within 1× NuPage transfer buffer. Afterwards, the membrane was blocked with 5% bovine serum albumin (BSA) in 1× PBS containing 0.1% Triton-X100. To analyze specific proteins, the membranes were incubated with following primary antibodies overnight: anti-UTX (Cell Signaling, Danvers, MA, USA, #33510S, 1:1000), anti-JMJD3 (Cell Signaling, #3457S 1:1000), anti-Histone H3 (clone: D1H2, Cell Signaling, #4499T, 1:2000), anti-H3K27me3 (Active Motif, Carlsbad, CA, USA, #039158, 1:5000), and, as loading control, anti-β-actin (#8H10D10). After washing, the membranes were incubated with secondary anti-mouse or anti-rabbit antibody (Cell Signaling, #3700, 1:1000; GE Healthcare, #NA931, 1:10,000 or GE Healthcare, #NA934, 1:10,000). For protein detection, SuperSignal™ West Dura Extended Duration Substrate (Thermo Scientific, Waltham, MA, USA) was added. The chemiluminescence signals were imaged with Fusion FX EDGE (Vilber, Marne-la-vallée, France) and automatically analyzed with ImageJ software (win-64, Fiji.app, accessed on 28 of April 2019).

### 2.13. Flow Cytometry

Cells were harvested with Accutase (Sigma-Aldrich) and stained either with the Aldefluor assay (Stem cell technologies) according to the manufacture’s recommendation or in PBS buffer with 5% FBS, 1% HEPES and 0.5 mM EDTA with anti-human CD44 (1:100, clone: IM7, #48-0441-80, Thermo Fisher) and anti-CD133 (1:50, clone: TMP4, #130133186, eBioscience). To exclude dead cells from the analysis, cells were additionally stained with the viability dye 7-Aminoactionomycin D (1:1000, Thermo Fisher). The samples were acquired with the BD FACSCelesta and analyzed with FlowJo software (version 10.9.0). The ALDH^+^ population was gated based on background fluorescence from the negative control, the diethylaminobenzaldehyde (DEAB) treated cells. The other CSC markers were gated according to the isotype controls (IgG-eFluor 450, eBioscience, #48-403182; rat anti-mouse IgG1-PE, eBioscience, #130-113-200).

### 2.14. RNA Sequencing

RNA was isolated after GSK-J1 treatment cells using the RNeasy mini kit (Qiagen; #74104) according to the manufacturer’s recommendation, in triplicate. The concentrations were measured with the NanoDrop ND-1000 (Peqlab). RNA sequencing was performed at the Genomics and Proteomics Core Facility (GPCF, DKFZ, Heidelberg, Germany). Libraries were prepared using the Illumina TruSeq Stranded Total RNA Library Prep Kit following the manufacturer’s instructions. Libraries concentration and quality control were performed using Qubit (Invitrogen) and Tapestation (Agilent). The samples were sequenced in a 2 × 100 bp paired-end setting on an Illumina HiSeq 4000 system according to the manufacturer’s protocol. The RNAseq raw data was demultiplexed, aligned, and mapped to genes to generate a raw count table following the DKFZ/ODCF RNAseq workflow (version 1.3.0-2). Down-stream data processing and statistical analysis were performed using DeSeq2 (version 1.30.1) in R 4.0.3 [21] excluding genes with low count number (<one count) from further analysis. Raw feature counts were transformed by the variance-stabilizing transformation (vst) and subsequent visualization with the ggplot2 R package (version 3.3.3, https://ggplot2.tidyverse.org, accessed on 4 January 2021). Functional enrichment and pathway analysis were carried out, with the genes having a Log2 fold change >2 and adjusted *p*-value < 0.05 in gProfiler (https://biit.cs.ut.ee/gprofiler/gost).

### 2.15. HNSCC Xenograft Model

Cal33 and FaDu xenografts were generated via subcutaneous transplantation of small pieces of tumors generated from a cryopreserved stock into the right hind leg of anesthetized NMRI (nu/nu) mice. Cisplatin (Calbiochem, Germany, 3 mg/kg b.w.) was dissolved in sodium chloride (0.9%) and administrated i.p. at the first day of treatment and then once weekly directly before irradiation as described by Koi et al. [22]. Control animals were injected with the same volume of sodium chloride. Local tumor irradiations were given with 18 and 12 Gy in 10 fractions for FaDu and Cal33, respectively. Tumors were excised 24 h after the last irradiation fraction for histological evaluation. The experimental procedure and the workflows within the animal facility followed the German animal welfare regulations and the ARRIVE guidelines and were approved by the institutional ethics committee (no: DD24-5131/207/34). 

### 2.16. Immunfluorescence Staining

Tumor tissues were sectioned into 7-µm-thick slices for immunofluorescence staining. Slides were fixed with methanol and acetone and washed with PBS. Unspecific binding was prevented via incubation in DAKO protein block (Agilent Dako, Santa Clara, California, USA) for 60 min at room temperature (RT). Primary antibodies against tri-methyl-histone H3 (#9733S, Cell Signaling), Histone H3 (#4499S, Cell Signaling), JMJD3 (#PA535012, Thermo Fisher Scientific) and UTX (#33510S, Cell Signaling) were diluted in 0.5% BSA in 1× PBS and incubated overnight at 4 °C in a humidified chamber. The samples were washed with 3% BSA in 1× PBS. Diluted Alexa Fluor 488 conjugated secondary antibodies were added and incubated for one hour at room temperature in the dark. Cell nuclei were stained with DAPI, and the samples were mounted with Mowiol mounting medium. Finally, the slides were scanned with a 20× magnification using a Zeiss Axio Scan.Z1 slide scanner (Zeiss, Jena, Germany).

### 2.17. In Silico Analysis of HNSCC Patient Data

*KDM6A/B* expression was investigated in tumor specimens of HNSCC patients that had received adjuvant radio/chemotherapy (RCTx). This independent explorative, retrospective study was conducted by the German Cancer Consortium Radiation Oncology Group (DKTK-ROG) and published by Lohaus et al. [23]. Ethical approval for clinical and biological data was obtained from the Ethics Committees of all DKTK partner sites. Briefly, 221 patients with locally advanced HNSCC received post-operative radio/chemotherapy (RCTx) based on cisplatin (81.6%) or mitomycin C (18.4%) between 2005 and 2011. For 187 out of 221 patients, tissue was available for gene expression profiling with the Affymetrix HTA2.0 platform. Patients were stratified according to a median threshold defined with the maxstat function (log-rank method) with the endpoints loco-regional control (LRC) to illustrate radiotherapy-specific local relapse, overall survival (OS), and distant metastasis (DM). Kaplan–Meier survival curves, log–rank statistics and hazard ratio were calculated with the GraphPad Prism software (version 8). The validation was performed on available data from the retrospective HNSCC cohort (*n* = 137) of the DKTK-ROG that received primary RCTx [24], including univariable and multivariable Cox regression analysis (Supplementary Table S1). The parameter age, tumor localization, HPV16 DNA, HPV16 RNA, P16 protein, p53 protein, *EZH2*, *HIF1A*, *KDM5B,* and *KDM6A* were included for multivariate analysis. Clinical and research data management was managed with the open-source software RadPlanBio (v1.0.0.14, 10.5281/zenodo.8298621,cacess date: 29 August 2023), a radiation/dose/plan, image/biomarker, and outcome platform (RPB).

### 2.18. Statistics

Radiobiological experiments were performed at least in three technical replicates, partially containing additional technical replicates depending on the readout. Statistical analyses to test for significant differences between experimental groups were performed using either a two-tailed *t*-test or ANOVA, depending on the experimental design, using SPSS (v23, IBM^®^, Chicago, IL, USA) or GraphPad Prism software (version 8, LLC Company, Boston, MA, USA). A one-tailed *t*-test was used for the Epigenetics Library Screen. A *p*-value < 0.05 was considered statistically significant and marked with *. Data are illustrated as violin plots including individual values or mean values including error bars as standard deviation (SD) or standard error of the mean (SEM) as indicated.

## 3. Results

### 3.1. Epigenetic Alterations in Radioresistant HNSCC Cells

**Figure 1:**
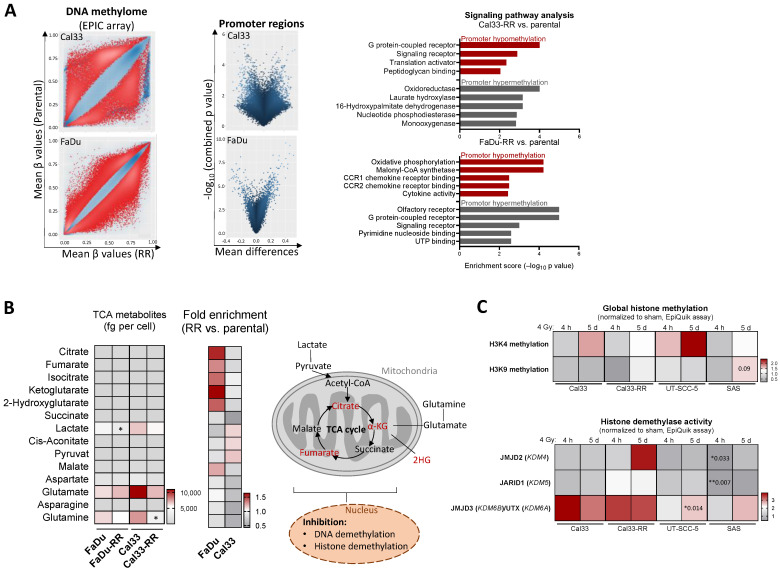
Epigenetic and metabolic alterations in radioresistant HNSCC cells. (**A**) EPIC array analysis to investigate global DNA methylation in radioresistant (RR) FaDu and Cal33 cells (*n* = 3). Genes with significantly altered DNA methylation status (red values) within the promoter region were included in the pathway analysis. Promoter hypomethylation (red) with active gene expression is shown separately from affected pathways with promoter hypermethylation (grey) and inhibited gene expression. (**B**) MS/MS-based analysis of Krebs cycle metabolites in parental and radioresistant (RR) Cal33 and FaDu cells (*n* = 3). The illustrative heatmaps show the concentration of the analyzed metabolites in femtogram (fg) per cell (left) as well as fold change with normalized data of RR to parental (right, * *p* < 0.05). The illustrative graphics highlight the metabolites with a higher concentration in RR populations that additionally function as co-factors for enzymes in epigenetic regulation. Abbreviation: 2HG 2-hydroxyglutarate, α-KG α-ketoglutarate) (**C**) EpiQuick assay to screen for alterations in global histone methylation of H3K4 and H3K9 as well as for histone demethylase (HDM) activities of JMJD2, JARID1, and JMJD3/UTX 4 h and 5 days after treatment with 4 Gy in different HNSCC cell lines. The data are shown as fold change normalized to untreated control (sham) (* *p* < 0.05; ** *p* < 0.01).

To understand the underlying molecular mechanisms driving irradiation-induced cellular plasticity in HNSCC, we performed DNA methylome and citrate cycle metabolome analyses to screen for alterations within radioresistant FaDu and Cal33 populations in comparison to their isogenic non-irradiated parental controls. Comparative DNA methylation profiling revealed profound alterations in the radioresistant Cal33 and FaDu populations. This may at least partially explain the enrichment of cells with intrinsic and acquired radioresistance during the long-term selection procedure. Reduced promoter methylation, indicative of an activated transcription, was found for genes involved in G-protein signaling in radioresistant Cal33 cells compared to the parental counterparts and for oxidative phosphorylation and chemokine receptor signaling in radioresistant FaDu cells (Figure 1A). 

As enzymes involved in DNA and histone methylation use metabolic co-factors provided by the tricarboxylic acid (TCA), we analyzed the intracellular concentration of different TCA metabolites. We found decreased level of lactate in radioresistant (RR) FaDu cells (*p* = 0.029, fold change = 0.66) and decreased intracellular glutamine concentrations in radioresistant Cal33 cells (*p* = 0.028, fold change = 0.54) compared to parental controls. Interestingly, Cal33 and FaDu cells are metabolically distinct: Citrate (*p* = 0.009), fumarate (*p* = 0.02), and α-ketoglutarate (*p* = 0.037) concentrations are significantly higher in Cal33 (Figure 1B).

Additionally, the global histone methylation levels of histone 3 lysine 4 (H3K4) increased 5 days after irradiation with 4 Gy in Cal33 and UT-SCC-5 cells, whereas the more resistant Cal33-RR and SAS cells did not show alterations. The permanent repressive histone mark H3K9 was increased in response to irradiation only in SAS cells (Figure 1C). 

Histone modifications are tightly regulated, e.g., through methylation site-specific writer, reader, and eraser enzymes. We analyzed the histone demethylase (HDM) activity of JMJD2, which targets H3K9 and H3K36, the H3K4-specific JARID1 and JMJD3, as well as the H3K27-specific UTX. Particularly, the JMJD3/UTX HDM activity was up-regulated 4 h and 5 days after irradiation with 4 Gy in all four tested HNSCC cell lines (*p* = 0.075 and *p* = 0.003 respectively, Figure 1C). Additionally, we analyzed H3K27me3 and H3K4me3 levels in a panel of six HNSCC cell lines with different radiosensitivities and found a positive correlation (Supplementary Figure S1A). 

In summary, we discovered profound alterations in epigenetic gene signatures, DNA methylation profiles within promoter regions, and HDM activity in radioresistant HNSCC cells compared to parental controls. 

### 3.2. Cellular Plasticity and Epigenetic Alterations in ALDH^+^ HNSCC CSCs

**Figure 2:**
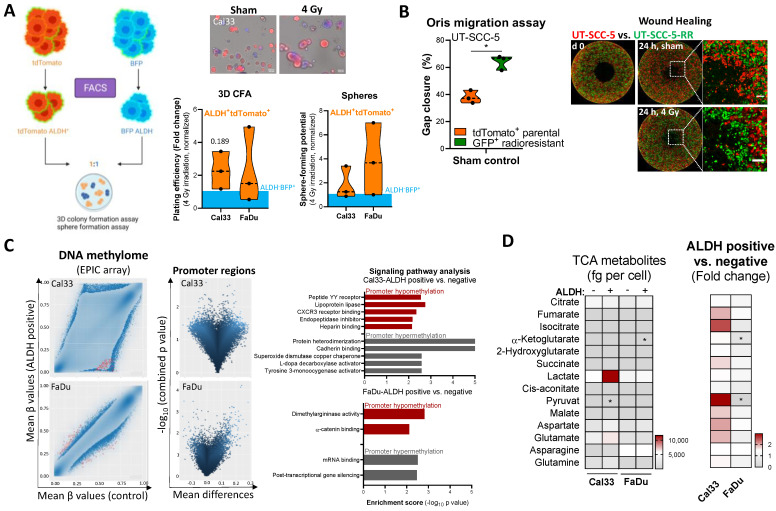
Cellular plasticity within the ALDH^+^ CSC population is determined via epigenetic and metabolic adaptation. (**A**) Competitive color-coding experiments were conducted to analyze the survival advantage of ALDH^+^ CSCs in Matrigel-based 3D and sphere cultures. After FACS purification, tdTomato^+^ALDH^+^ cells were mixed in a 1:1 ratio with BFP^+^ALDH^−^ cells (*n* = 3, Cal33 and FaDu, scale bar 100 μm). (**B**) Oris-based competitive cell migration assay combining a mixture of tdTomato-expressing parental UT-SCC-5 in a 1:1 ratio with GFP-expressing UT-SCC-5-RR cells. The gap closure was evaluated 24 h after irradiation with 4 Gy and compared to sham control (*n* = 3, scale bar 200 μm, * *p* < 0.05). The dotted white square marks the region of the magnified image on the right side. (**C**) Global DNA methylation was evaluated in ALDH^+^ CSCs compared to the ALDH^−^ control in Cal33 and FaDu cells using the EPIC array (*n* = 3). Genes with a differentially methylated promoter region were included in pathway analysis. An activated gene expression is characterized by promoter hypomethylation, whereas promoter hypermethylation is a characteristic of repressed genes. (**D**) Metabolites of the tricarboxylic acid (TCA) cycle were analyzed after fluorescence-activated cell sorting (FACS)-based purification of 50,000 ALDH^+^ and ALDH^−^ Cal33 and FaDu cells (*n* = 3). The data are illustrated as intracellular concentrations in femtogram (fg) per cell and as fold change for ALDH^+^ CSCs normalized to ALDH^−^ controls.

To investigate the mechanisms governing the irradiation-induced enrichment of radioresistant CSCs, we performed a competitive co-culture analysis, to demonstrate a clonogenic survival advantage of ALDH^+^ over ALDH^−^ cultures. For this analysis, Cal33 and FaDu cells were labeled with blue (BFP) and red fluorescence protein (tdTomato). To exclude survival advantages stemming from the fluorescent transgene, these cell lines were characterized for their proliferative, clonogenic potential in 2D and 3D and their sphere forming capacity as well as expression of *ALDH1A3* (Supplementary Figure S2A–D). After purification of tdTomato^+^ALDH^+^ cells, these cells were mixed in a 1:1 ratio with BFP^+^ALDH^−^ cells, plated for 3D colony and sphere formation assays, and irradiated to assess radiosensitivity and self-renewal capabilities. The colony and sphere forming capacity of ALDH^+^ CSCs showed a nonsignificant increase in both cell lines after irradiation with 4 Gy (*p* = 0.108 and *p* = 0.112 respectively, Figure 2A) as well as in controls with a swapped transgene (Supplementary Figure S2E,F). Additionally, we investigated the competitive migratory behavior of GFP-expressing, radioresistant UT-SCC-5 cells in comparison to the tdTomato-positive parental control using a gap closure-based assay (Oris). UT-SCC-5-RR cells had a higher migratory capacity than parental cells. No differences were seen upon 4 Gy irradiation (Figure 2B). 

To investigate the epigenetic and metabolic landscape of ALDH^+^ CSCs, we performed a multi-OMIC screening and compared the signatures to non-CSC bulk cultures. When comparing DNA methylation between ALDH^+^ CSCs and negative control cells, we found only marginal differences, particularly compared to the dramatic changes seen in the radioresistant population. Differentially hypomethylated promoter regions in Cal33 ALDH^+^ CSCs were associated with an activated transcription of genes involved in lipoprotein lipase, CXCR3 receptor binding, or heparin binding signaling, whereas genes repressed by promoter hypermethylation were associated with cadherin binding, superoxide dismutase and L-dopa decarboxylase signaling pathways (Figure 2C). We assume that clinically relevant biomarker can be identified within the intersection of radioresistant and ALDH^+^ CSCs. Therefore, we compared the differential gene expression of radioresistant and ALDH^+^ Cal33 and FaDu cells. We identified nine common genes *(DENND2C, KCNJ18, DAD1, RUNX3, TOX4, FLJ32255, NPTX1, VPS13C, HSP90AA1*) belonging to the signaling pathways of has-miR-148a-3p, CTP binding, and dATP binding (Supplementary Figure S1B). The same was done for the DNA metabolome datasets. We identified 2101 DNA sequences with altered DNA methylation in radioresistant and ALDH^+^ CSCs belonging to pathways involved in lauric acid metabolism, linoleic acid metabolism, nuclear fragmentation and apoptotic change, malonate catabolic process, and eicosanoid biosynthesis (Supplementary Figure S1C).

Additionally, we found increased intracellular concentrations of the TCA metabolites fumarate (*p* = 0.039), malate (*p* = 0.098), and aspartate (*p* = 0.043) in ALDH^+^ CSCs compared to the ALDH^−^ controls in combined values from Cal33 and FaDu cell lines. A cell line-specific increase was found for pyruvate in ALDH^+^ Cal33 cells and a decrease of α-ketoglutarate in ALDH^+^ FaDu cells (Figure 2D). 

These findings collectively demonstrate epigenetic and metabolic alterations in ALDH^+^ CSCs in comparison to HNSCC bulk cultures that may impact cellular sensitivity, survival, and plasticity after irradiation.

### 3.3. High Throughput Screening for Epigenetic Targeting Compounds with Radiosensitizing Potential in HNSCC Cells

**Figure 3:**
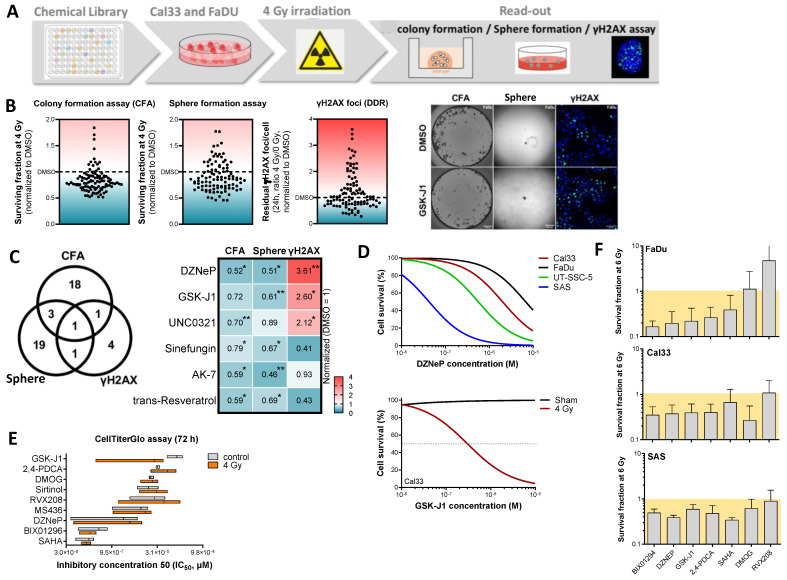
Epigenetic library screen to identify compounds with radiosensitizing and CSC-targeting effects in HNSCC cell lines. (**A**) Schematic illustration of the experimental screening setup including cell plating, treatment, and readout of clonogenicity after 10 days, DNA repair capacity with the γH2AX assay after 24 h, and the self-renewal potential with the sphere formation assay after 14 days. The HNSCC cell lines FaDu and Cal33 were seeded in 96-well plates followed by 24 h of pre-treatment with 5 μM of the Epigenetics Screening Library (Cayman Chemical, cat. no. 11076), consisting of 146 compounds before irradiation with 4 Gy (*n* = 2). (**B**) Combined analysis of screening results obtained from FaDu and Cal33 cell were normalized to DMSO and shown as fold change. Out of 146 compounds, 23 (15.8%) exhibited radiosensitizing effects in the colony formation assay (CFA) readout, 24 compounds (16.4%) affected self-renewal, and 7 compounds affected DNA damage repair (DDR) after irradiation (4.8%) (*n* = 4, * *p* < 0.05, one-sided paired *t*-test). Compounds with plating efficiency < 0.05 in CFA were regarded as cytotoxic and eliminated from further analysis. (**C**) The illustrative heatmap summarizes the six identified compounds with a significant radiosensitizing effect in at least two readouts. The mean values are normalized to vehicle control. The positive control 3-deazaneplanocin A (DZNeP), an inhibitor of S-adenosylmethionine-dependent methyltransferase that targets the degradation of EZH2, has the highest radiosensitizing potential. GSK-J1 targets the H3K27me3- and H3K4me3/2/1-specific demethylases UTX andJMJD3, UNC0321 the histone methyltransferase G9a, Sinefungin is a natural analog of s-adenosyl-L-methionine (SAM) and is a pan-DNA methyltransferase (DNMT) inhibitor. AK-7 targets the ADP-ribosyl transferase and deacetylase SIRT2 (sirtuin), and trans-resveratrol is a polyphenolic natural compound found in grapes and berries (* *p* < 0.05; ** *p* < 0.01). (**D**) Nine epigenetic targeting compounds with high radiosensitizing effects in the screening setup were further validated in the HNSCC cell lines Cal33, FaDu, UT-SCC-5, and SAS. For this, the cells were treated for 72 h with increasing concentrations of the inhibitors to determine the half maximal inhibitory concentration (IC_50_) with the CellTiterGlo-based cell viability assay. The exemplary curves show the results for the EZH2 inhibitor DZNeP and the JMJD3/UTX inhibitor GSK-J1. (**E**) The cell viability and IC_50_ assay were also combined with 4 Gy irradiation after a one-hour pre-treatment with the compounds. Data are presented as mean IC_50_ values of the pooled five HNSCC cell lines (*n* = 3). (**F**) Six out of nine previously selected compounds were further tested using 3D colony formation assay. The shown data are normalized to sham and DMSO control (*n* = 3, mean ± SD).

To evaluate epigenetic targeting as a strategy for radiosensitization of HNSCC, we conducted a chemical library screen with 146 compounds in combination with photon irradiation in FaDu and Cal33 cell lines. After plating, cells were treated with epigenetic modifiers at a concentration of 5 μM and irradiated with a single dose of 4 Gy. Subsequently, we assessed DNA damage repair capacity using the γH2AX assay, as well as clonogenic and spherogenic survival, to determine radiosensitizing and CSC-targeting potential (Figure 3A,B, Supplementary Figure S3A–D). The screening was performed twice for both cell lines and readouts. 

Of the 146 compounds included in the library, 23 compounds (15.8%) significantly decreased clonogenic survival, 24 compounds (16.4%) significantly decreased spherogenic survival, and 7 compounds (4.8%) significantly impaired DNA damage repair as indicated by γH2AX foci compared to DMSO control. In total, 6 compounds (4.1%) exhibited a significant radiosensitizing effect in two or more readouts. Notably, we found therapeutic potential in the EZH2 inhibitor 3-Deazaneplanocin A (DZNeP), which displayed a significant effect in all readouts. Furthermore, we identified GSK-J1 as a promising radiosensitizing compound in HNSCC cells. Other promising hits included UNC0321, sinefungin, AK-7, and trans-resveratrol (Figure 3C). 

We further compared and combined the screening data from HNSCC cells with previously obtained data for prostate cancer (PC, DU145 and PC3 cells) [19] and glioblastoma (GBM, LN229 and U-87 MG) [20], identifying 16 compounds with radiosensitizing potential in all three tumor entities with at least two readouts (Supplementary Figure S3C,D). DZNeP and GSK-J1 were among the compounds with the highest radiosensitizing potential. 

Hits from the screen were further validated in the four HNSCC cell lines Cal33, FaDu, UT-SCC5, and SAS, using cell proliferation and 3D colony formation assays. For this purpose, we determined the half maximal inhibitory concentration (IC_50_) of nine selected inhibitors 72 h after treatment (Figure 3D). Based on these values, we calculated the IC_5_ values for further application. Additionally, we combined this analysis after one-hour inhibitor treatment with 4 Gy irradiation and confirmed the high radiosensitizing potential of GSK-J1 (Figure 3D,E). Except for the TET3-inhibitor dimethyloxalylglycine (DMOG) and the bromodomain and extra-terminal protein inhibitor (BETi) Apabetalone (RVX-208), we confirmed the radiosensitizing potential for all other five compounds after one-hour pre-treatment at concentrations equal to the IC_5_ value (Figure 3F). 

These findings underscore the potential of combining epigenetic targeting with irradiation to enhance radiotherapy efficacy in HNSCC.

### 3.4. Histone Demethylase Inhibitor GSK-J1 Alters Radiosensitivity of HNSCC Cells

**Figure 4:**
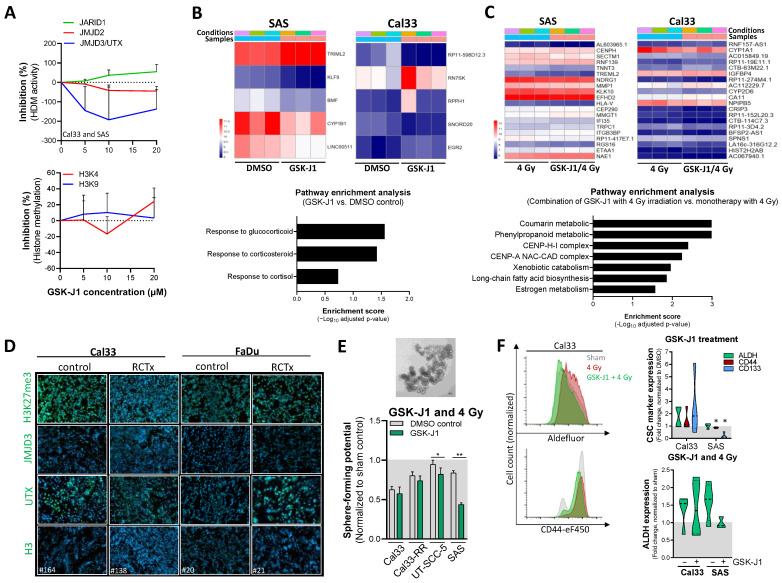
Radiobiological investigation of GSK-J1 treatment in combination with irradiation in HNSCC cells. (**A**) The EpiQuick assay was performed 24 h after treatment of Cal33 and SAS cells with increasing doses of GSK-J1 to investigate the inhibitory effects and target specificity for the histone demethylases (HDM) JMJD2 (*KDM4*), JARID1 (*KDM5*), and UTX (*KDM6A*)/JMJD3 (*KDM6B*) as well as global histone H3K4 and H3K9 methylation (combined values; *n* = 1 per cell line). (**B**) Differential gene expression analysis with RNA sequencing for Cal33 and SAS cells 24 h after treatment with GSK-J1 monotherapy in comparison to the DMSO control. The heatmap shows significantly differentially expressed genes as log2 ratios that were further used for pathway enrichment analysis (graph below). (**C**) The same analysis was performed for the combination of GSK-J1 with 4 Gy irradiation in comparison to the treatment with irradiation alone to investigate GSK-J1-specific molecular mechanisms of radiosensitization (*n* = 3 each, combined values for Cal33 and SAS). (**D**) Immunofluorescence analysis to investigate H3K27me3 intensity as well as JMJD3 and UTX protein expression (green) in tissue sections derived from Cal33 and FaDu xenograft tumors that were s.c. transplanted into NMRI nu/nu mice (nuclear DAPI counterstaining). Established xenograft tumors were treated with a cisplatin-based chemotherapy one hour before fractionated irradiation (5 fractions per week, 6 weeks). The tumors were harvested when the endpoint of maximal tumor size was reached (100 µm^2^). (**E**) Sphere formation after GSK-J1 treatment in combination with 4 Gy irradiation to determine the inhibitory effect on self-renewal (*n* = 3, mean ± SD, * *p* < 0.05, ** *p* < 0.01). (**F**) Flow cytometry-based analysis to determine the expression of putative CSC markers such as aldehyde dehydrogenase (ALDH), CD133, and CD44 in Cal33 and SAS cells 7 days after GSK-J1 treatment (IC_5_, 4.5 µM) in combination with 4 Gy irradiation (*n* = 3, * *p* < 0.05).

We validated the specificity of GSK-J1 in the HNSCC cell lines FaDu, Cal33, and SAS and measured the histone demethylation activity of JMJD3/UTX at increasing GSK-J1 concentrations. As a control, we compared the effects to the H3K9 and H3K36 methylation-specific HDM JMJD2 and the H3K4m3-specific HDM JARID1. The mean JMJD3/UTX activity was decreased by 192.6% at 10 µM GSK-J1. However, the observed inhibitory effect was cell line specific: 20.7% in Cal33 cells and 253.9% in SAS cells. No inhibitory effects of GSK-J1 were found on JARID1 or JMJD2 activity, nor on H3K4 or H3K9 methylation (Figure 4A). 

To investigate the underlying molecular mechanisms involved in GSK-J1-mediated radiosensitization, we analyzed the transcriptome of GSK-J1-resistant SAS and GSK-J1-sensitive Cal33 cells 24 h after GSK-J1 treatment (IC_5_, 4.5 µM). We found five differentially regulated genes in Cal33 after GSK-J1 monotherapy and no enriched signaling pathway, whereas SAS cells exhibited an altered response to cortisol, glucocorticoids, and corticosteroids (Figure 4B). In SAS cells, the combination of GSK-J1 with 4 Gy of irradiation altered genes involved in the centromeric nucleosome-associated complex CENP and other components of the centromeric chromatin, which are responsible for nucleosome formation. In Cal33 cells, the majority of altered signaling pathways were involved in cellular metabolism, such as coumarin, xenobiotic catabolism, long-fatty acid biosynthesis, and estrogen metabolism (Figure 4C). In vivo, we found reduced H3K27me3 and UTX protein levels in Cal33-derived subcutaneous xenograft tumors after radio/chemotherapy (RCTx) in comparison to control tumor sections, whereas the opposite effect was seen in FaDu-derived tumors (Figure 4D). 

Moreover, we investigated the effects of GSK-J1 treatment on adhesion-independent cell growth in growth factor-defined media and analyzed the sphere forming potential. We found that the combination of GSK-J1 with 4 Gy irradiation decreased the stem-like potential compared to the DMSO control in all tested cell lines (*p* = 0.005, Figure 4E). Additionally, the GSK-J1 treatment reduced the expression of putative CSC markers such as CD44 and CD133 in SAS cells (Figure 4F). 

These data indicate a heterogenous response of different HNSCC cell lines, populations, and tumors to GSK-J1 treatment. 

### 3.5. Histone Demethylases JMJD3 and UTX as Regulators for Radiation Sensitivity in HNSCC Cells 

**Figure 5:**
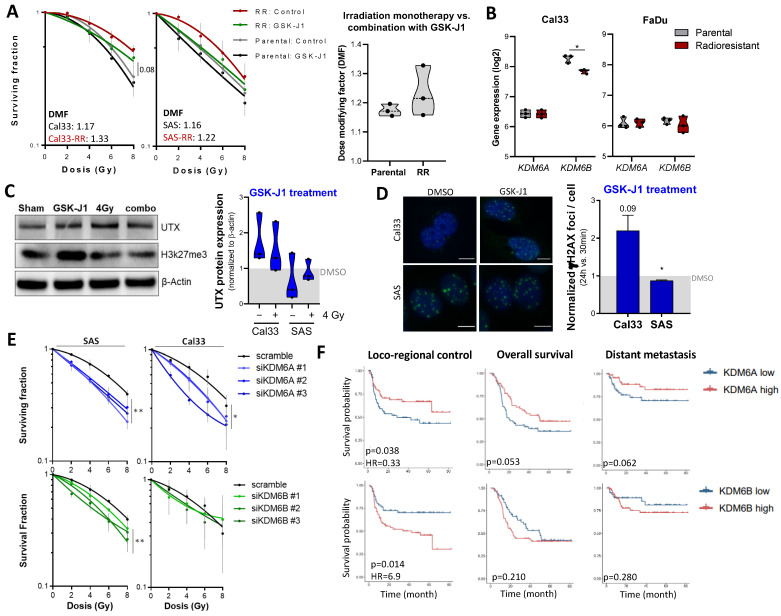
Radiobiological characterization of the identified compound GSK-J1 in HNSCC. (**A**) Parental (grey and black curves) and radioresistant (RR, red and green curves) Cal33 and SAS cells were plated as single-cell suspensions in Matrigel. After pre-treatment with the mean IC_5_ concentration of GSK-J1 (4.5 µM) for one hour, the cells were irradiated, and formed colonies were counted after 10 days to determine the clonogenic survival and radiosensitizing effects under GSK-J1 treatment. The survival curves were generated based on the linear–quadratic model to calculate curve differences (LQ model, *n* = 3, mean ± SD). The dose-modifying factor (DMF) for GSK-J1 indicates a dose reduction value compared to the DMSO control group with the same biological effect at 4 Gy. (**B**) Gene expression of the GSK-J1 targets *KDM6A* (UTX) and *KDM6B* (JMJD3) in parental and radioresistant Cal33 and FaDu cells (*n* = 3, * *p* < 0.05). (**C**) Western blot to illustrate GSK-J1 and 4 Gy irradiation effects on H3K27 trimethylation and the protein expression of the histone demethylase UTX in Cal33 cells (*n* = 3). The pixel quantifications of the band signals were normalized to the loading control β-actin. The data are shown as fold change normalized to the solvent DMSO control. (**D**) γH2AX foci assay to illustrate DNA double-strand break and repair 30 min (initial foci) after 4 Gy irradiation to illustrate the maximal damage, and 24 h (residual foci) to analyze the cellular DNA damage repair capacity in Cal33 and SAS cells. The GSK-J1 pre-treatment occurred one hour before irradiation with the IC_5_. The same amount of DMSO solvent was used as control (*n* = 3, mean ± SD, * *p* < 0.05). The data are shown as fold change with normalized residual to initial foci. (**E**) Genetic silencing of the GSK-J1 targets *KDM6A* (UTX) and *KDM6B* (JMJD3) via siRNA-mediated knock-down in Cal33 and SAS cells 24 h before plating the cells in Matrigel-based 3D cultures and subsequent irradiation. Formed colonies were evaluated 10 days after plating (*n* = 3, mean ± SD, LQ model, ** *p* < 0.01 and * *p* < 0.05). (**F**) Kaplan–Meier survival curves that separate HNSCC patients undergoing primary radio/chemotherapy (*n* = 137, DKTK cohort [23], Affymetrix array) according to the median expression of *KDM6A* (UTX) and *KDM6B* (JMJD3) in pre-treatment biopsies. The data were analyzed according to different clinical endpoints such as overall survival (OS), loco-regional control (LRC), and distant metastasis (DM) (Log–rank test, HR = hazard ratio) (Supplementary Table S1). Original western blots are presented in File S1.

To validate our screening results, we performed a Matrigel-based 3D clonogenic survival assay. Before irradiation, we pre-treated parental and radioresistant (RR) Cal33 and SAS cells with the mean 5% inhibitory concentration (IC_5_) of GSK-J1 (4.5 µM). The radiosensitizing effect was higher in the radioresistant population compared to the parental control, as calculated by the dose-modifying factor (DFM) (Figure 5A). 

To exclude the possibility that the differences seen between radiosensitive and -resistant populations were mediated via differential expression of *KDM6A* and *KDM6B*, we compared the gene expression and found a lower expression of *KDM6B* in Cal33-RR compared to the parental control (Figure 5B). With Western blot, we analyzed the UTX protein expression and H3K27 trimethylation of all four treatment groups in Cal33 and SAS cells. H3K27me3 increased in response to irradiation in Cal33 cells (fold change: 1.57), whereas the methylation level decreased in SAS cells (fold change: 0.53). Interestingly, cells treated with the combination of GSK-J1 and 4 Gy exhibited H3K27me3 levels comparable to the DMSO control (Figure 5C). 

Since cellular radiosensitivity depends on the DNA repair capacity, we analyzed DNA double-strand breaks after GSK-J1 treatment in combination with 4 Gy irradiation using the γH2AX assay. We did not observe alterations after GSK-J1 treatment alone and in combination with irradiation after 30 min, but we found delayed DNA damage repair in Cal33 cells 24 h after treating cells with the combination of GSK-J1 and 4 Gy compared to the DMSO control (Figure 5D). In addition to the chemical inhibition, we validated the radiosensitizing effect of the respective HDMs UTX (*KDM6A*) and JMJD3 (*KDM6B*) upon gene-specific knock-down. While knock-down of both HDMs resulted in lower survival fractions in SAS and Cal33 cells with more pronounced effects in SAS, this difference did not reach statistical significance (Figure 5E). The human epidermal keratinocyte cell line HaCaT, which was used as normal tissue control, did not show any GSK-J1 treatment effect (Supplementary Figure S4A). 

### 3.6. Prognostic and Therapeutic Potential of KDM6A and KDM6B in HNSCC Patients

In an available gene expression dataset from tumor specimens of HNSCC patients treated with primary radio/chemotherapy (*n* = 137, DKTK-ROG) [23], we analyzed the clinical potential of *KDM6A* (UTX) and *KDM6B* (JMJD3) as putative prognostic biomarkers and stratified patients into high and low expression groups based on median gene expression values. The analysis showed that high expression of *KDM6A* (UTX) is associated with high locoregional control (LRC), but no association could be found for overall survival (OS) and distant metastasis (DM). In contrast, high *KDM6B* expression showed a significantly negative prognostic value for OS (Figure 5F, Supplementary Table S1). 

In the analyzed cohort, 34% of the tumors were found to be human papilloma virus (HPV)-positive, which may affect the results, because it has been previously shown that the HPV E7 oncoprotein has the potential to induce *KDM6A* and *KDM6B* gene expression [25]. We stratified the cohort based on HPV status and analyzed the HPV-negative cases separately. Like in the cohort of patients treated with primary radio/chemotherapy, high *KDM6A* expression was associated with a more favorable prognosis, and high *KDM6B* expression was associated with a worse outcome, although this effect was not significant (Supplementary Figure S4B). 

The prognostic potential of *KDM6B* was validated in a cohort of HNSCC patients who received post-operative radio/chemotherapy (*n* = 187, DKTK-ROG cohort, [9]) (Supplementary Table S1). Additionally, we investigated the prognostic value of other well-known epigenetic regulators, such as *KDM5B*, *EZH2*, and *HDAC4*. We found that a high *KDM5B* and *HDAC4* expression identified patients with a worse response to radio/chemotherapy, whereas a high *EZH2* expression was indicative of a favorable prognosis (Supplementary Figure S4C–E, Supplementary Table S1). 

To conclude, we validated the putative therapeutic potential of GSK-J1 for radiosensitization of HNSCC cells and identified its targets UTX (*KDM6A*) and JMJD3 (*KDM6B*) as potential prognostic biomarkers in HNSCC patients undergoing radiotherapy. 

## 4. Discussion

Tumor heterogeneity and cellular plasticity have a significant impact on tumor progression, therapy resistance, and metastatic dissemination. This heterogeneity may arise from CSCs that can dynamically respond to environmental changes, therapeutic interventions, or immune cell attacks [26,27]. CSCs play a critical role in influencing tumor radiosensitivity, and achieving lasting tumor control necessitates the complete elimination of all CSCs [6,28]. However, an increased cellular plasticity observed during radiotherapy has been documented, which hinders the effective elimination of CSCs in various tumor types, including HNSCC [15,17,18,29]. CSC plasticity may result from epigenetic reprogramming [26] and involves histone modifications as well as DNA methylation [30,31,32,33]. 

In our study, we observed profound epigenetic and metabolic alterations in radioresistant HNSCC cells and ALDH^+^ CSCs. These findings led us to hypothesize that modulating the epigenetic landscape through the inhibition of epigenetic regulatory proteins could serve as a promising therapeutic strategy for radiosensitizing of HNSCC. To identify potential compounds, we conducted a high-throughput chemical library screening including various clinically relevant epigenetic inhibitors, which revealed six compounds with high radiosensitizing and CSC-targeting potential. One of these compounds is the histone H3K27 methyltransferase EZH2 inhibitor DZNeP, which we previously identified as a candidate that targets ALDH^+^ CSCs in prostate cancer [18]. The most promising new candidate we identified for HNSCC is the histone demethylase inhibitor GSK-J1, which targets UTX (*KDM6A*) and JMJD3 (*KDM6B*).

UTX is required for embryonic development and lineage-specific differentiation [34]. In HNSCC, UTX is frequently mutated and has been described as a metabolically regulated, X-linked tumor suppressor [35,36,37]. Moreover, the activity of the oxygen-sensitive UTX (*KDM6A*) is reduced under hypoxic conditions, whereas its paralog JMJD3 (*KDM6B)* remains unaffected [38]. Therefore, GSK-J1 may influence cancer cell radiosensitivity by influencing the oxidative stress response of cancer cells [39]. Another proposed inducer of JMJD3 (*KDM6B*) transcription is the canonical NOTCH signaling [40]. Down-stream of JMJD3, genes involved in response to cellular and environmental stresses, differentiation, proliferation, senescence, and apoptosis are positively regulated. Both UTX (*KDM6A*) and JMJD3 (*KDM6B*) are Jumonji motif-containing demethylases and dependent on α-ketoglutarate and Fe^2+^ for efficient enzymatic activity. The identified compound GSK-J1 is a competitive inhibitor and prevents the binding of both cofactors to JMJD3 and UTX. 

Chemosensitizing effects, reduced cell proliferation, and apoptosis induction have been previously shown for GSK-J1 in HNSCC cells. Zhang et al. combined GSK-J1 with TCP, an inhibitor of the H3K4me1/2-specific HDM LSD1 (*KDM1A*), and found synergistic effects [41]. Kruidenier et al. assessed the target specificity of GSK-J1 with an AlphaScreen assay and found inhibitory potency against JARID1B (*KDM5B*) and JARID1B (*KDM5C*) [42]. Additionally, GSK-J4, a pro-drug and ethyl ester derivative of GSK-J1, which penetrates cells more effectively and is hydrolyzed intracellularly to GSK-J1, demonstrated radiosensitizing effects in patient-derived xenograft models of diffuse intrinsic pontine glioma (DIPG). The authors noted a reduced expression of DNA repair genes and changes in DNA accessibility through ATAC sequencing after GSK-J4 treatment. This led to an increased number of sustained DNA double-strand breaks after irradiation, as demonstrated by the γH2AX and 53BP1 foci assay, as well as reduced clonogenic survival and increased survival of tumor-bearing mice treated with GSK-J4 in combination with radiotherapy [43]. Our study in HNSCC cell lines revealed similar findings, showing radiosensitizing effects of GSK-J1 in 3D clonogenic survival and γH2AX assays. Furthermore, we discovered that GSK-J1 treatment prior to irradiation prevents irradiation-induced plasticity, as indicated by reduced sphere forming potential and a decreased CSC marker expression. 

**Limitation:** The epigenetic inhibitor screen was carried out within 2D culture models. Those model systems have the disadvantage that they do not represent cell-cell and cell-ECM interactions. Therefore, we applied Matrigel-based 3D culture and sphere formation assays in liquid cultures because those model systems mimic cell interactions, cell morphology, microenvironment, oxygen, and nutrient gradients as existing in human tumors. The strengths of the performed epigenetic inhibitor screening is the read-out with three different biological endpoints for radiosensitivity, self-renewal and DNA repair to identify compounds targeting several pathways involved in radioresistance. The screening approach could be improved in the future through the addition of different compound concentrations and treatment time points. Moreover, a validation of our findings in murine models would be necessary before clinical translation.

**Outlook:** The identification of UTX (*KDM6A*) as putative therapeutic target and/or potential prognostic biomarker for HNSCC patients undergoing radiotherapy draws our attention to the currently upcoming discussion about the importance of X- and Y-linked tumor suppressor genes and/or oncogenes influencing sex-specific cancer incidence and prognosis [44]. UTX (*KDM6A*) is located on the X chromosome and carries sex-specific mutations associated with an increased cancer incidence in males [45,46]. Within our study, we used the HNSCC cell lines Cal33 and FaDu, which originate from male patients, whereas SAS was derived from a female HNSCC patient. These sex differences may influence the sensitivity to GSK-J1 treatment. Further projects investigating these findings are needed to implement them within clinical workflows and improve cancer therapy efficiency through sex-specific prognosis and treatment. 

## 5. Conclusions

We identified GSK-J1 as a potent epigenetic inhibitor that specifically targets the histone demethylases UTX (*KDM6A*) and JMJD3 (*KDM6B*) with synergistic potential in HNSCC cells when combined with irradiation. We found that both GSK-1J targets differ in their prognostic effects for HNSCC patients undergoing radio-/chemotherapy, pointing to an ambiguous function of GSK-J1 treatment in combination with radiotherapy and underscoring the importance of patient stratification for assessing treatment susceptibility. 

## Data Availability

The DNA methylation (Figure 1A and Figure 2C) and RNASeq data (Figure 4C,D) can be downloaded from the GEO repository (GSE254171). The data of the retrospective DKTK-ROG cohort is available through the corresponding authors of Linge et al. [28].

## Supplementary Materials

The following supporting information can be downloaded at: https://www.mdpi.com/article/10.3390/cancers16040730/s1, Supplementary Figure S1. Radioresistance gene signature in HNSCC cells is upstream regulated by epigenetic mechanisms, Supplementary Figure S2. Evaluation of putative molecular and cellular effects of the fluorescence color-code in the HNSCC cells, Supplementary Figure S3. High-throughput epigenetic library screen for compounds with radiosensitizing and CSC-targeting potential in cell lines of different tumor entities, Supplementary Figure S4. Prognostic value of the GSK-J1 targets UTX (*KDM6A*) and JMJD3 (*KDM6B*) in a cohort of HNSCC patients undergoing radiotherapy, Supplementary Table S1. Univariable and multivariable Cox regression analysis of UTX (*KDM6A*) and JMJD3 (*KDM6B*) transcript level in pre-treatment biopsies of HNSCC patients that received adjuvant or primary radio/chemotherapy (RCTx) and were included in the German Cancer Consortium Radiation Oncology Group (DKTK-ROG) study initially published by Lohaus et al. [23] and Linge et al. [24], and Supplementary Table S2. DNA sequences with differential DNA methylation in promoter regions of radioresistant and ALDH^+^ CSC Cal33 and FaDu cells. File S1: Original western blots.
