# Supplementary material for: Epigenetic Targeting to Overcome Radioresistance in Head and Neck Cancer
Source: Cancers (Basel). 2024 Feb 9;16(4):730. doi: 10.3390/cancers16040730 (PMC10886471; doi:10.3390/cancers16040730)
Supplement: Supplementary file 1 [file cancers-16-00730-s001.zip › cancers-2776602-supplementary.pdf]

Article

Epigenetic Targeting to Overcome Radioresistance in Head and Neck Cancer

Iñaki Schniewind [1,2,8], Maria José Besso [6], Sebastian Klicker [1], Franziska Maria Schwarz [2,3,4], Wahyu Wijaya Hadiwikarta [6], Susan Richter [7], Steffen Löck [2,5], Annett Linge [1,2,3,5], Mechthild Krause [1,2,3,4,5], Anna Dubrovska [1,2,3,4], Michael Baumann [2,6], Ina Kurth [6] and Claudia Peitzsch [1,2,9,*]

Supplementary data

Supplementary Table S1. Univariate and multivariate Cox regression analysis for UTX (*KDM6A*) and JMJD3 (*KDM6B*) transcript level in specimen of HNSCC patients that received post-operative (n = 187) or primary (n = 137) radio/chemotherapy (RCTx). The German Cancer Consortium Radiation Oncology Group (DKTK-ROG) study results with the primary endpoint locoregional control (LRC) was initially published by Lohaus et al. [1] and Linge et al. [2]. Multivariate Cox regression analysis was only performed for markers with significance in univariate calculation and include the parameters age, tumor localization, HPV16 DNA, HPV16 RNA, p16 protein, p53 protein, *EZH2*, *HIF1A*, *KDM5B* and *KDM6A* (HR, hazard ratio, *p < 0.05, **p < 0.01).

	Post-operative cohort				Primary cohort			
	Univariate Cox		Multivariate Cox		Univariate Cox		Multivariate Cox	
	HR	p	HR	p	HR	p	HR	p
KDM6A	0.03	0.013*	0.04	0.070	0.33	0.290		
KDM6B	0.89	0.930			6.90	0.029*	3.80	0.200
KDM5B	6.70	0.005**	2.97	0.230	2.00	0.130		
EZH2	0.12	0.009**	0.65	0.650	0.67	0.400		
HDAC4	38.0	0.077			6.10	0.200		

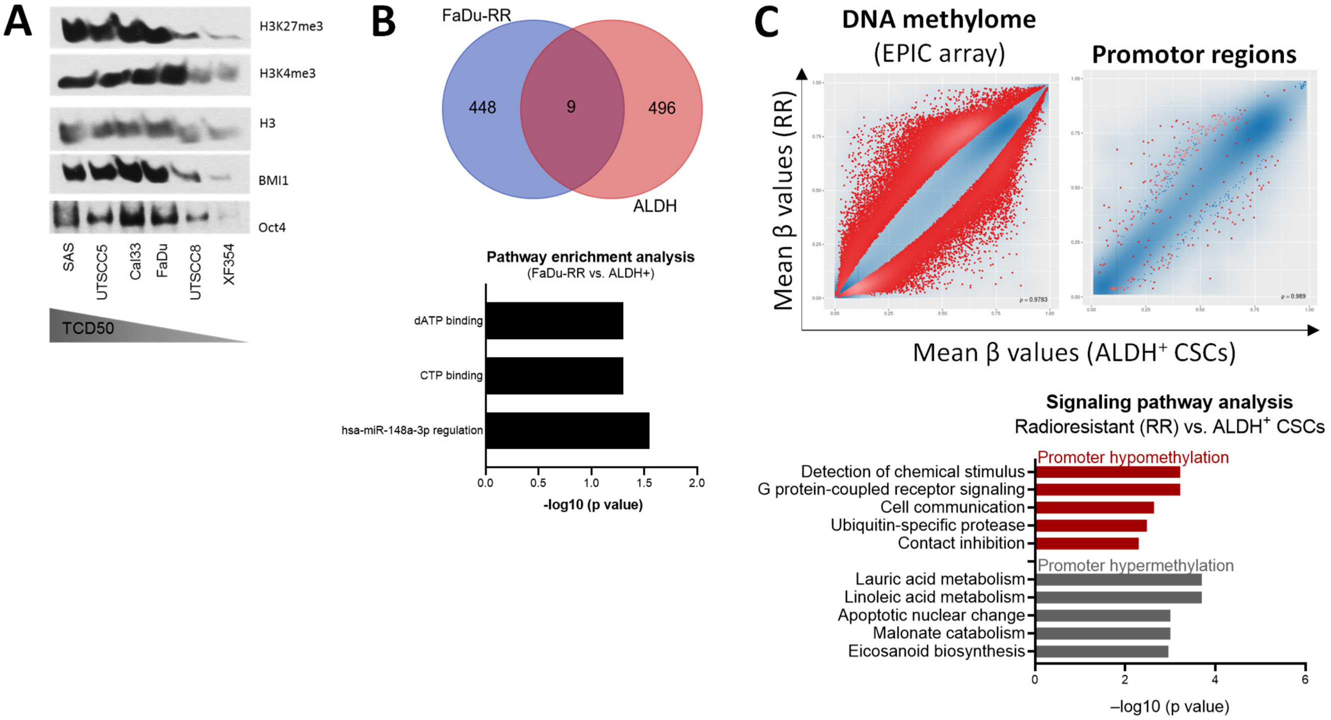

Supplementary Figure S1. Radioresistance in HNSCC cells is upstream regulated by epigenetic mechanisms. A) Western blot analysis comparing histone H3K27 and H3K4 tri-methylation and the CSC-specific transcription factors BMI1 and Oct4 in HNSCC cells ordered according to their cell intrinsic radiosensitivity based on experimentally obtained tumor control dose 50% (TCD50) values [3–5]. **B)** Comparative gene expression analysis of the intersection between radioresistant (RR) and ALDH$^+$ CSCs from FaDu cells identified nine common genes that are involved in the dATP binding, CTP binding, and has-miR-148a-3p regulation ($n = 3$, $p > 0.05$). **C)** Comparative DNA methylome of the intersection between radioresistant (RR) and ALDH$^+$ CSCs of FaDu and Cal33 cells. The analysis focused on DNA methylation pattern within the promoter region and identified signaling pathways with active gene transcription (promoter hypomethylation) and deactivated genes (promoter hypermethylation) ($n = 3$, $p < 0.05$).

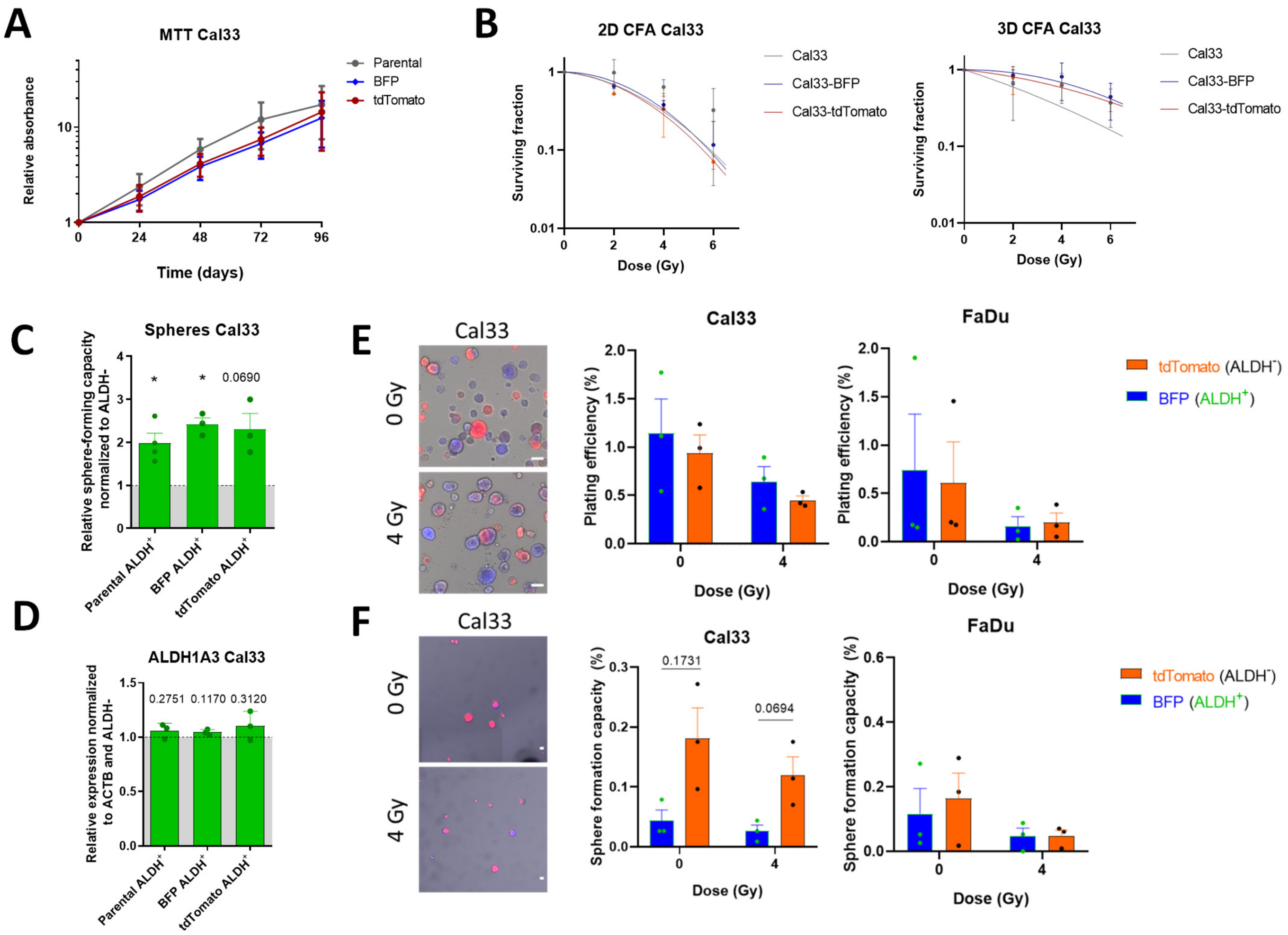

Supplementary Figure S2. Evaluation of putative molecular and cellular effects of the fluorescence color-code in the HNSCC cells. A) Cell proliferation was analyzed in Cal33 cell expressing the blue fluorescence protein (BFP) and tdTomato in comparison to the parental control with MTT assay (n = 3 to 5, mean ± SD). **B)** The radiosensitivity of BFP- and tdTomato-expressing Cal33 cells was analyzed with a conventional 2D and 3D colony formation and compared to the parental control (n = 2, mean ± SD). **C)** ALDH+ CSCs from Cal33 cells were FACS-purified and the sphere formation capacity analyzed (n = 3, mean ± SD, two-sided unpaired t-test, *$p < 0.05$). **D)** The *ALDH1A3* gene expression was analyzed in FACS-purified ALDH+BFP+ and ALDH+tdTomato+ Cal33 cells and compared to the parental control (n = 3, mean ± SD, two-sided unpaired t-test). **E)** Competitive 3D clonogenic and **F)** spherogenic survival assay of BFP+ALDH+ CSCs mixed in a ratio 1:1 with tdTomato+ALDH- control cells in Cal33 and FaDu (n = 3, mean ± SEM, two-sided paired t-test, scale bar 100 µm).

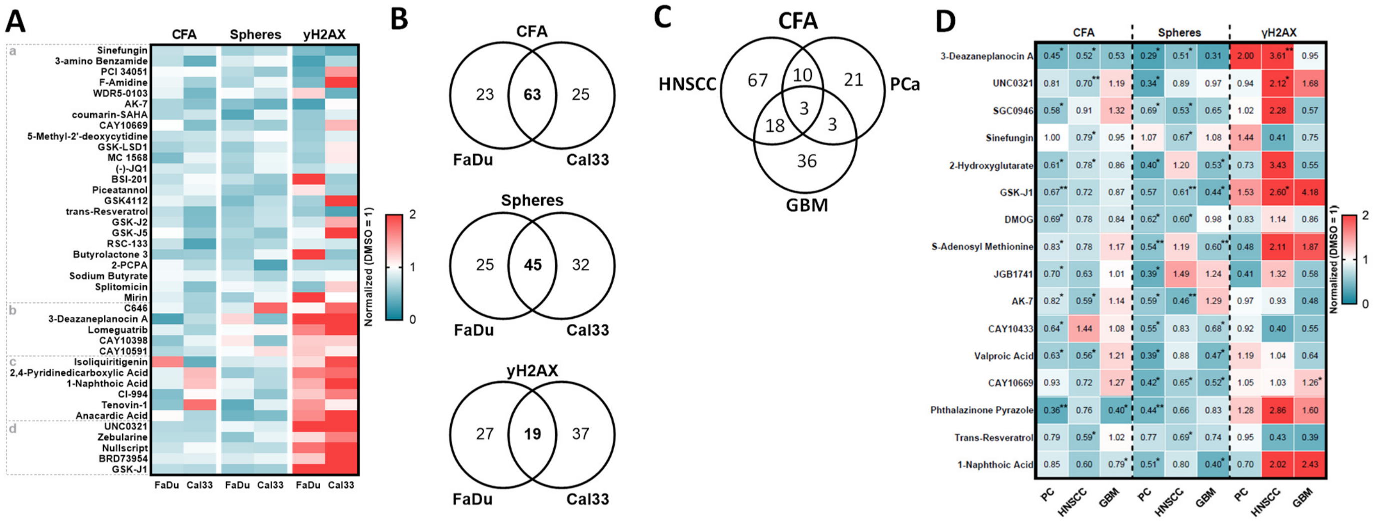

Supplementary Figure S3. High-throughput epigenetic library screen for compounds with radiosensitizing and CSC-targeting potential in cell lines of different tumor entities. A) Cal33 and FaDu HNSCC cells were plated as single cell suspension in 96-well plate 24 hours before treatment with the Epigenetics Screening Library (5 µM, Cayman Chemical, cat. no. 11076) that consists of 146 compounds. 24 hours later, the plates were irradiated with 4 Gy and the clonogenic survival was evaluated 10 days after treatment, the sphere-forming potential after 14 days and the DNA repair with the γH2AX assay after 24 hours (n = 2). The heatmap shows fold change values normalized to the solvent DMSO control. For the shown compounds, significant results in at least one cell line and two readouts were obtained. The compounds are ranked according to their radiosensitizing and CSC-targeting potential. **B)** Venn diagrams summarizing the screening results to compare the different response in the two tested cell lines FaDu and Cal33 within the three different readout assays. **C)** The obtained results for the epigenetic screening library in the two HNSCC cell lines FaDu and Cal33 were compared to the previously published results in the two prostate cancer (PC) cell lines DU145 and PC3 [6], and glioblastoma (GBM) cell lines LN229 and U87-MG [7]. **D)** Heatmap including compounds with a significant radiosensitizing effect in at least two readouts of one or more entities. The data obtained by the two cell lines were combined and the shown values are fold changes normalized to the DMSO control (n = 4, *$p < 0.05$, **$p < 0.01$, one-sided paired t-test). Compounds with plating efficiency lower than 0.05 within the colony formation assay (CFA) were regarded as cytotoxic and eliminated from further analysis.

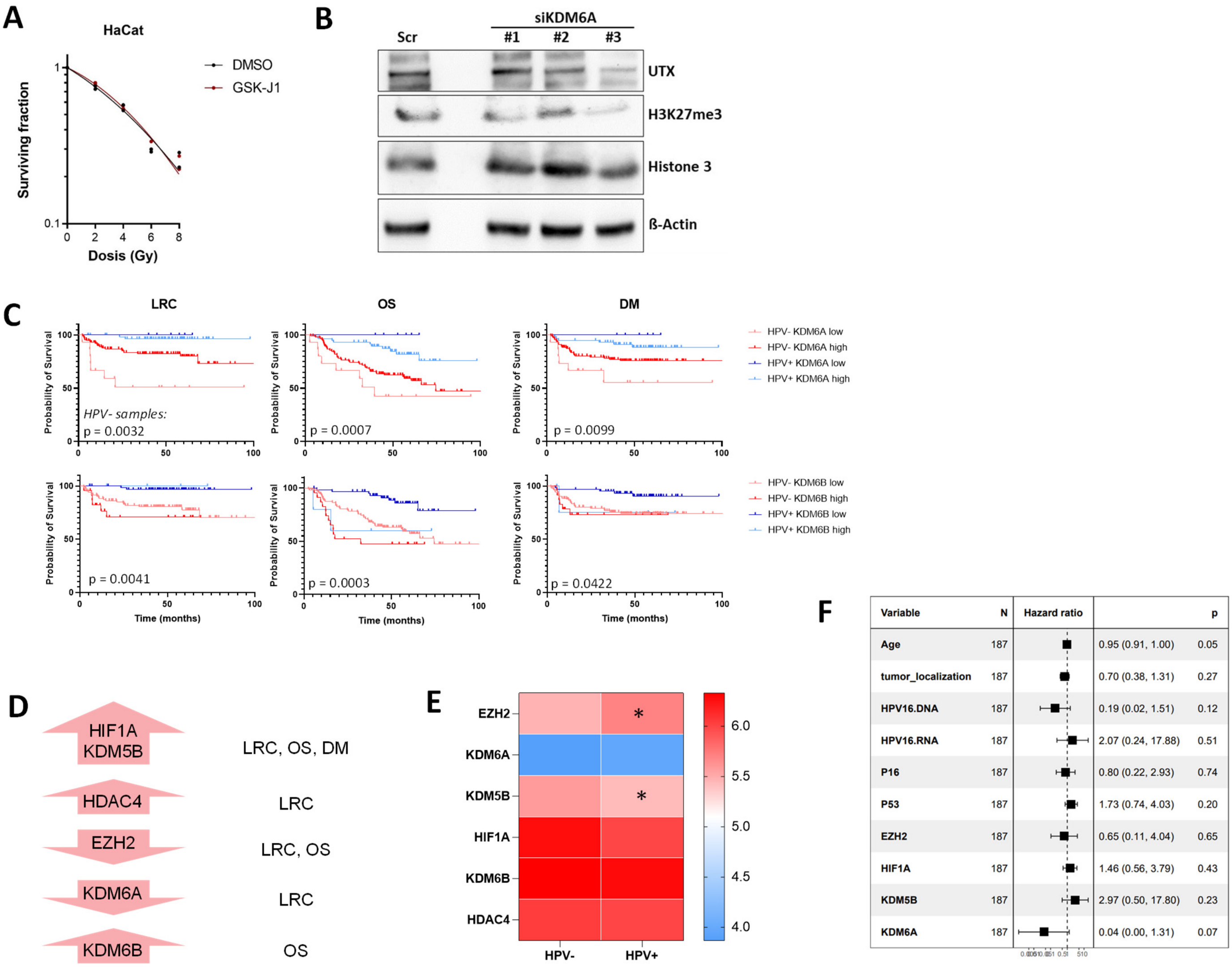

Supplementary Figure S4. Prognostic value of the GSK-J1 targets UTX (*KDM6A*) and JMJD3 (*KDM6B*) in a cohort of HNSCC patients undergoing radiotherapy. A) Putative normal tissue toxicity of GSK-J1 in combination with irradiation was tested in the immortalized keratinocyte cell line HaCaT with the 3D clonogenic survival assay. Curves are adjusted according to the linear-quadratic model (n = 2, mean). **B)** Western blot to validate the KDM6A (UTX) knock-down on protein level and on the down-stream target, the H3K27 trimethylation, after 48 hours in Cal33 (n = 2). **C)** HNSCC patients undergoing post-operative radio/chemotherapy (n=196, DKTK cohort, Affymetrix array) were separated according to their papilloma virus (HPV) status (positive: 34%) and according to median expression of UTX (*KDM6A*) and JMJD3 (*KDM6B*) in pre-treatment biopsies. Kaplan-Meier survival curves are shown according to different clinical endpoints such as overall survival (OS), loco-regional control (LRC), and distant metastasis (DM) (Log-rank test). **D)** Illustrative summary of the obtained clinical results and putative stratifying biomarkers. **E)** The heatmap shows the stratifying potential of the investigated markers in HPV-positive and -negative HNSCC patients separately. The color indicates the gene expression level in pre-treatment biopsies that are characterized by worse patient outcome after radiotherapy (blue: low, red: high gene expression level. **F)** Forest plot including the parameter for multivariable analysis of the HNSCC patients treated with post-operative radio/chemotherapy such as age, tumor localization, HPV16 DNA, HPV16 RNA, P16 protein, p53 protein, EZH2, HIF1A, KDM5B, and KDM6A.

References

1. Lohaus, F.; Linge, A.; Tinhofer, I.; Budach, V.; Gkika, E.; Stuschke, M.; Balermpas, P.; Rödel, C.; Avlar, M.; Grosu, A.-L.; et al. HPV16 DNA Status Is a Strong Prognosticator of Loco-Regional Control after Postoperative Radiochemotherapy of Locally Advanced Oropharyngeal Carcinoma: Results from a Multicentre Explorative

Study of the German Cancer Consortium Radiation Oncology Group (DKTK-ROG). *Radiother Oncol* **2014**, *113*, 317–323, doi:10.1016/j.radonc.2014.11.011.

2. Linge, A.; Lohaus, F.; Löck, S.; Nowak, A.; Gudziol, V.; Valentini, C.; von Neubeck, C.; Jütz, M.; Tinhofer, I.; Budach, V.; et al. HPV Status, Cancer Stem Cell Marker Expression, Hypoxia Gene Signatures and Tumour Volume Identify Good Prognosis Subgroups in Patients with HNSCC after Primary Radiochemotherapy: A Multicentre Retrospective Study of the German Cancer Consortium Radiation Oncology Group (DKTK-ROG). *Radiotherapy and Oncology* **2016**, *121*, 364–373, doi:10.1016/j.radonc.2016.11.008.
3. Helbig, L.; Koi, L.; Brüchner, K.; Gurtner, K.; Hess-Stumpp, H.; Unterschemmann, K.; Baumann, M.; Zips, D.; Yaromina, A. BAY 87-2243, a Novel Inhibitor of Hypoxia-Induced Gene Activation, Improves Local Tumor Control after Fractionated Irradiation in a Schedule-Dependent Manner in Head and Neck Human Xenografts. *Radiat Oncol* **2014**, *9*, 207, doi:10.1186/1748-717X-9-207.
4. Koi, L.; Bergmann, R.; Brüchner, K.; Pietzsch, J.; Pietzsch, H.-J.; Krause, M.; Steinbach, J.; Zips, D.; Baumann, M. Radiolabeled Anti-EGFR-Antibody Improves Local Tumor Control after External Beam Radiotherapy and Offers Theragnostic Potential. *Radiother Oncol* **2014**, *110*, 362–369, doi:10.1016/j.radonc.2013.12.001.
5. Koi, L.; Löck, S.; Linge, A.; Thurow, C.; Hering, S.; Baumann, M.; Krause, M.; Gurtner, K. EGFR-Amplification plus Gene Expression Profiling Predicts Response to Combined Radiotherapy with EGFR-Inhibition: A Preclinical Trial in 10 HNSCC-Tumour-Xenograft Models. *Radiother Oncol* **2017**, *124*, 496–503, doi:10.1016/j.radonc.2017.07.009.
6. Schwarz, F.M.; Schniewind, I.; Besso, M.J.; Lange, S.; Linge, A.; Patil, S.G.; Loeck, S.; Klusa, D.; Dietrich, A.; Voss-Boehme, A.; et al. Plasticity within Aldehyde Dehydrogenase-Positive Cells Determines Prostate Cancer Radiosensitivity. *Mol Cancer Res* **2022**, molcanres.MCR-21-0806-A.2021, doi:10.1158/1541-7786.MCR-21-0806.
7. Schniewind, I.; Hadiwikarta, W.W.; Grajek, J.; Poleszczuk, J.; Richter, S.; Peitzsch, M.; Müller, J.; Klusa, D.; Beyreuther, E.; Löck, S.; et al. Cellular Plasticity upon Proton Irradiation Determines Tumor Cell Radiosensitivity. *Cell Rep* **2022**, *38*, 110422, doi:10.1016/j.celrep.2022.110422.
