# Supplementary material for: Epigenetic Targeting to Overcome Radioresistance in Head and Neck Cancer
Source: Cancers (Basel). 2024 Feb 9;16(4):730. doi: 10.3390/cancers16040730 (PMC10886471; doi:10.3390/cancers16040730)

Western Blot (Figure 5C)

SAS

1. Sham
2. Sham+GSKJ1
3. IR
4. IR+GSKJ1

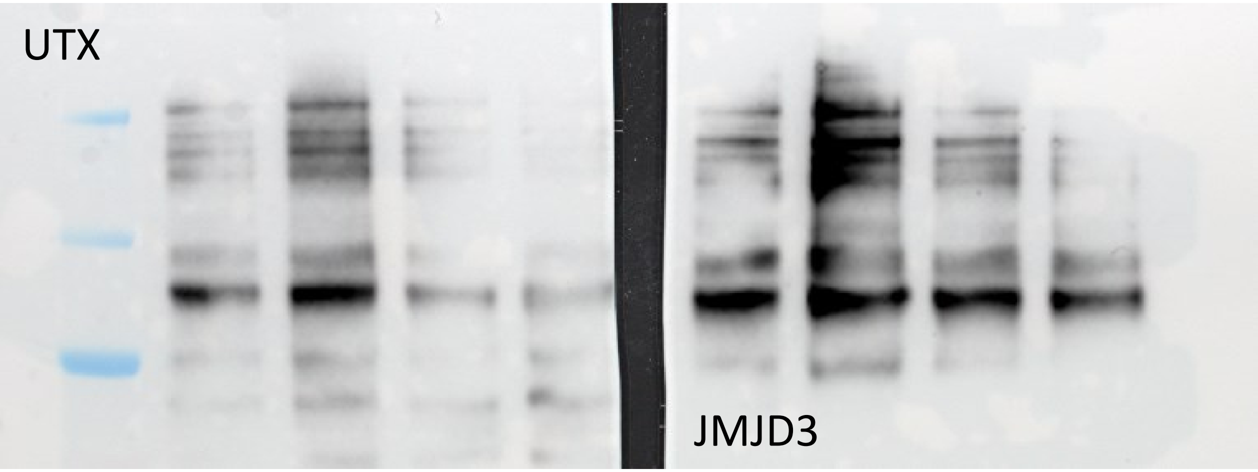

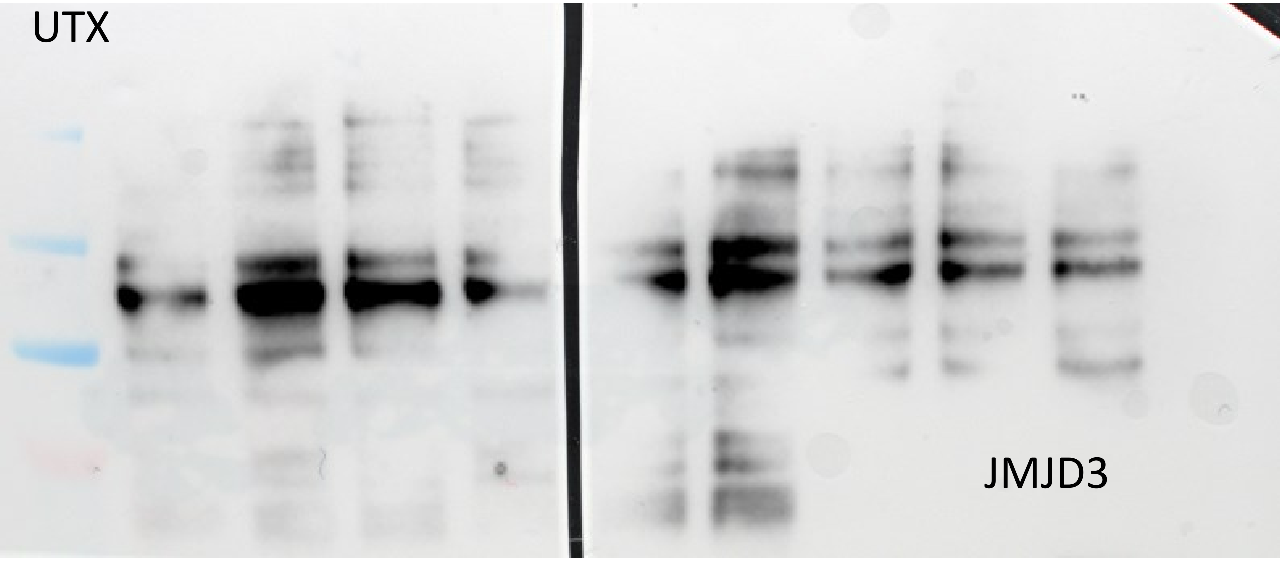

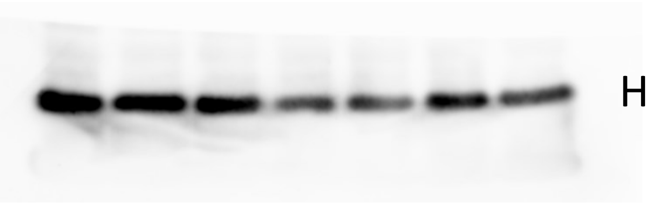

H3K27me3

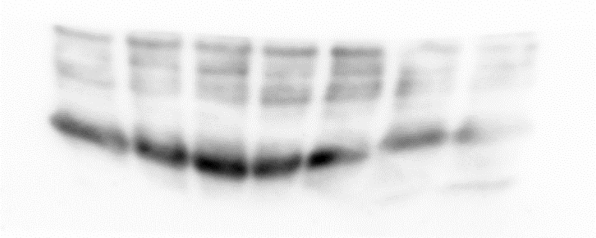

H3

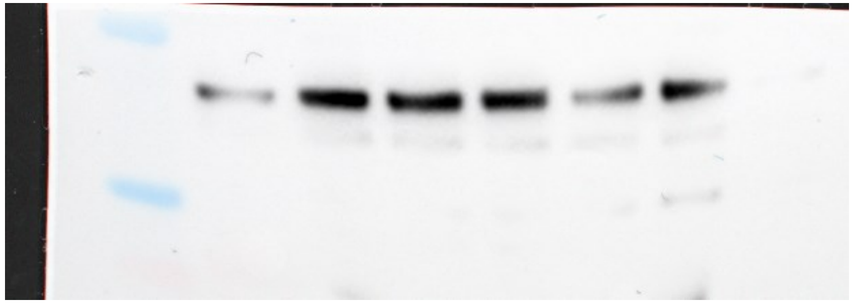

ß-Actin

Western Blot (Figure 5C)

Cal33, n1&2

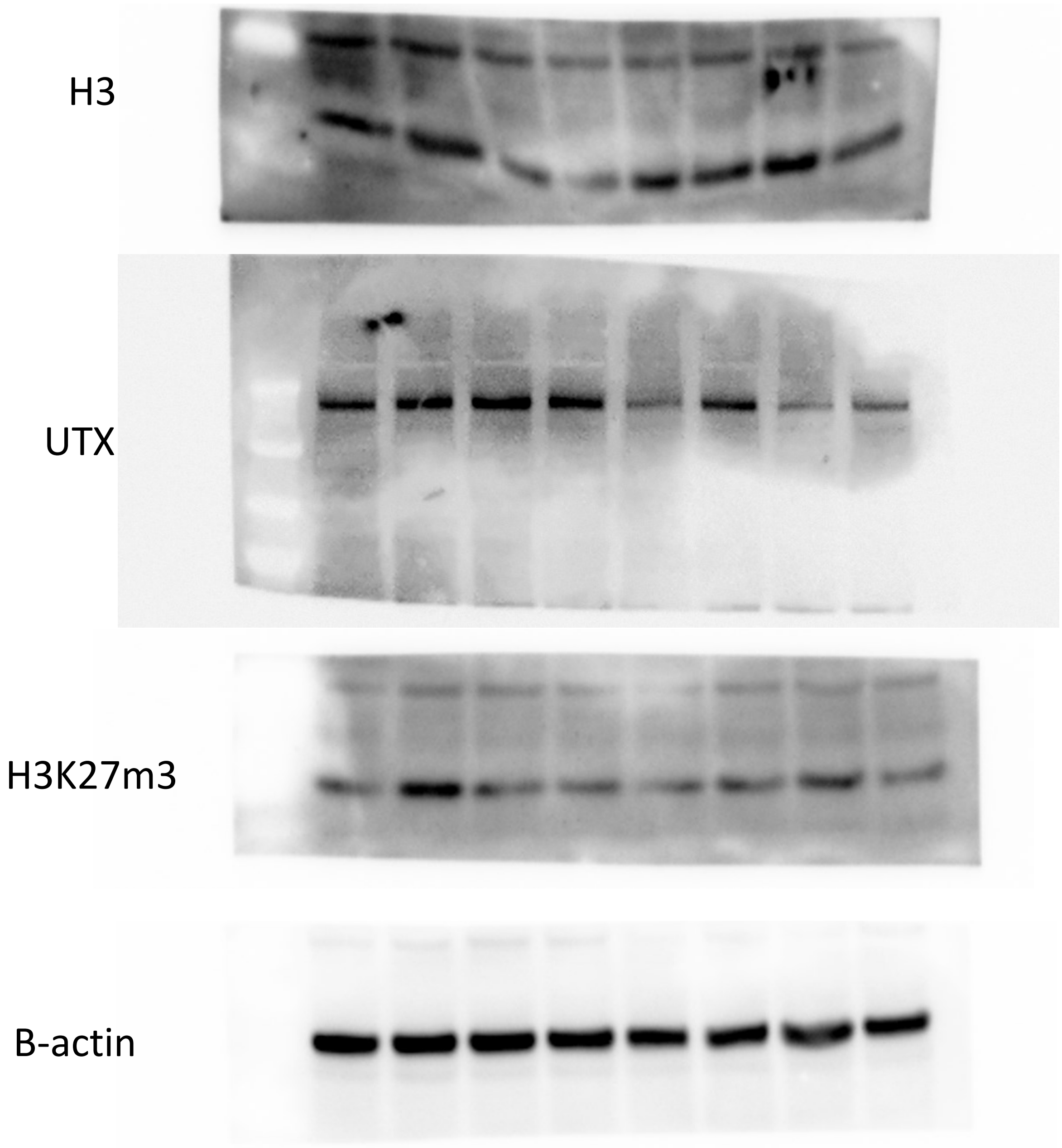

Western Blot (Figure 5C)

n=3 Cal33+SAS

H3

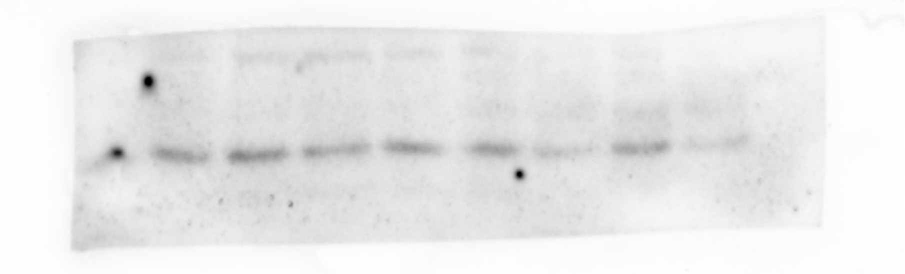

UTX

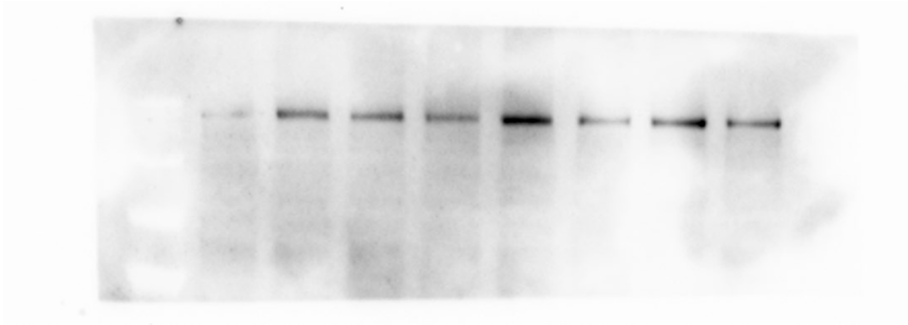

H3K27m3

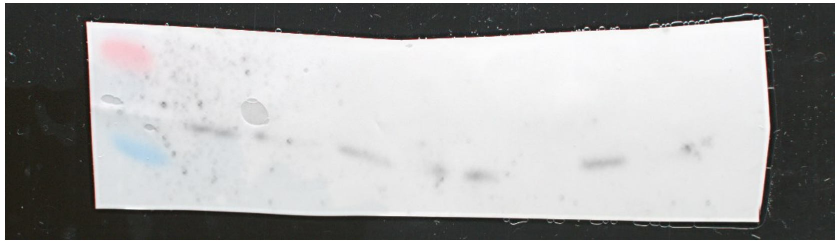

B-actin

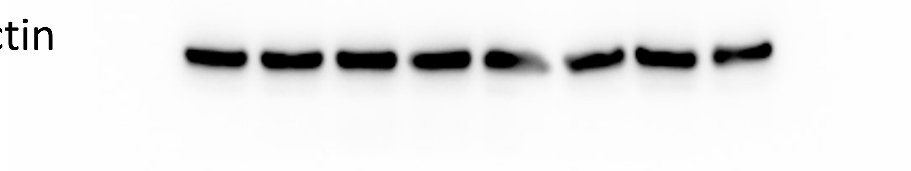

Western Blot (Figure 5C)

SAS, n2, milk

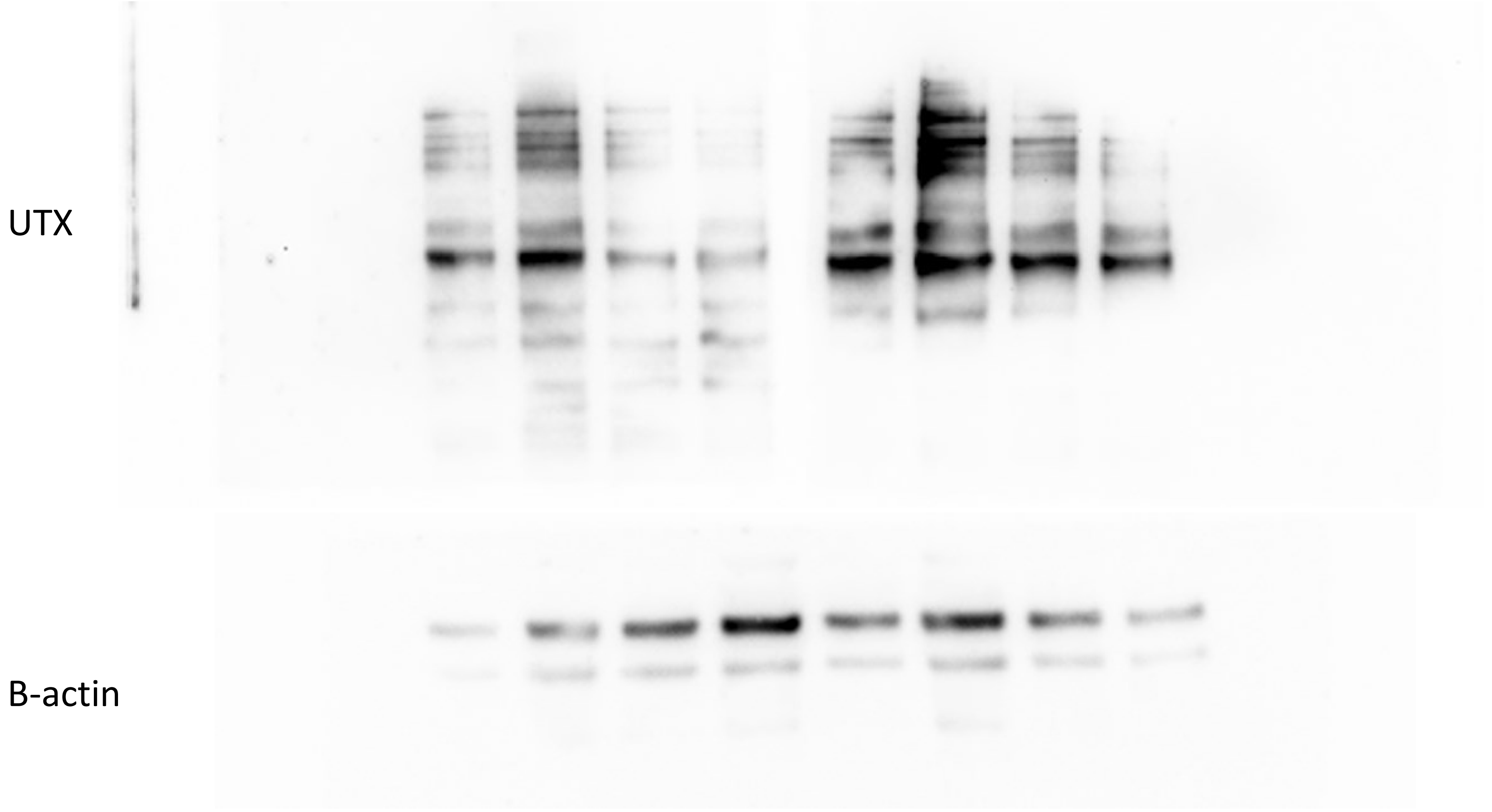

Western Blot (Supplementary Figure S4B)

Cal33 (Lane5)

Lane: 1,2,3 = KDM6A

Lane: 7,8,9 = KDM6B

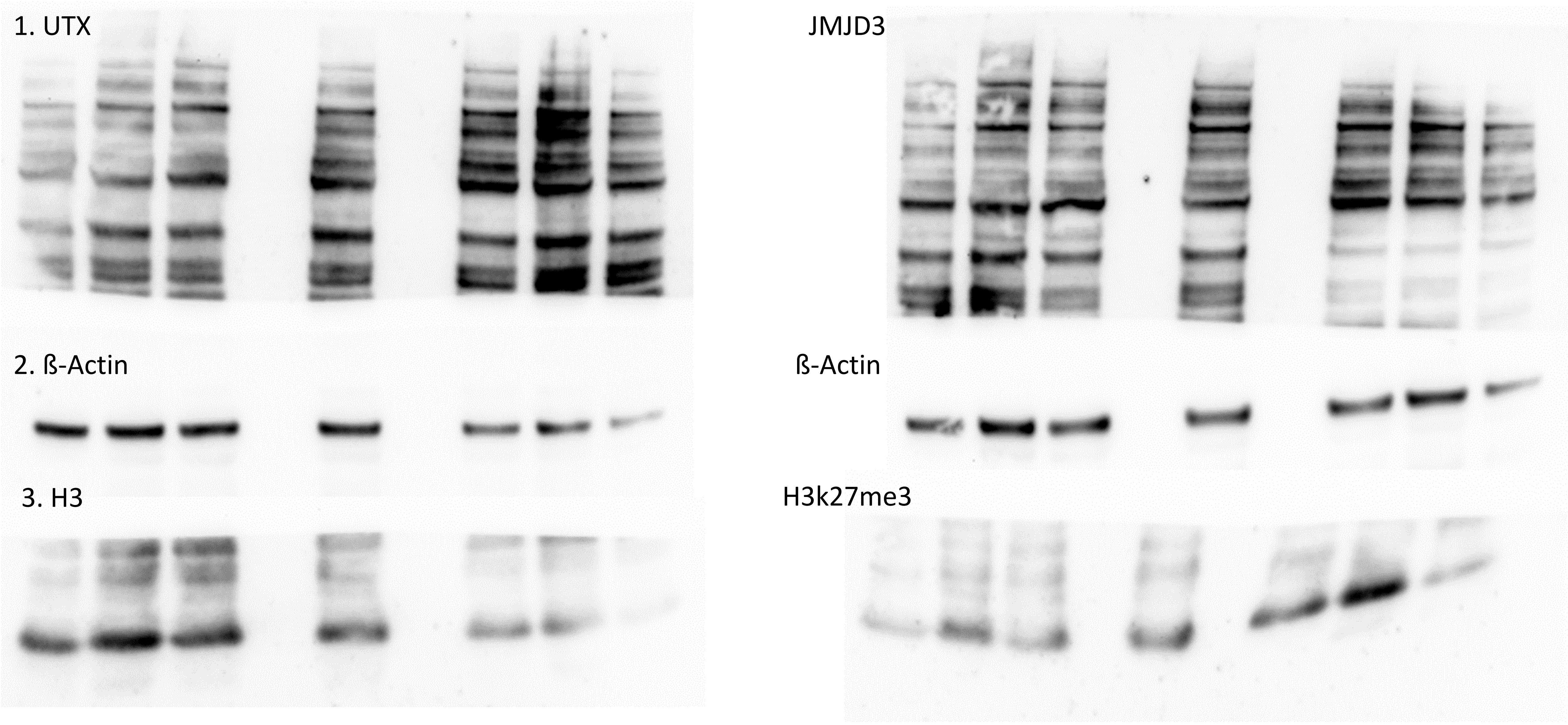

Western Blot (Supplementary Figure S4B)

Cal33 (Lane5)

Lane: 1,2,3 = KDM6A

Lane: 7,8,9 = KDM6B

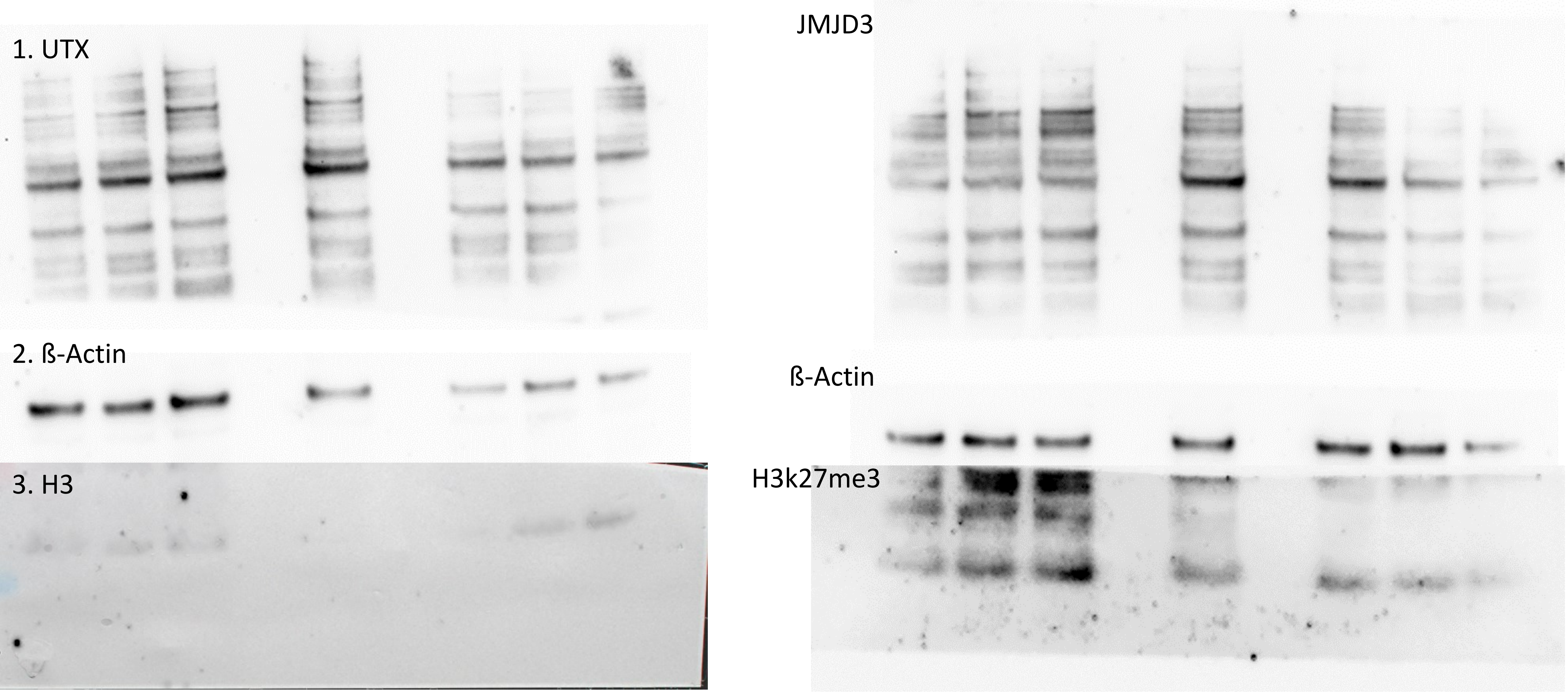

Western Blot (Supplementary Figure S4B)

Cal33, n1

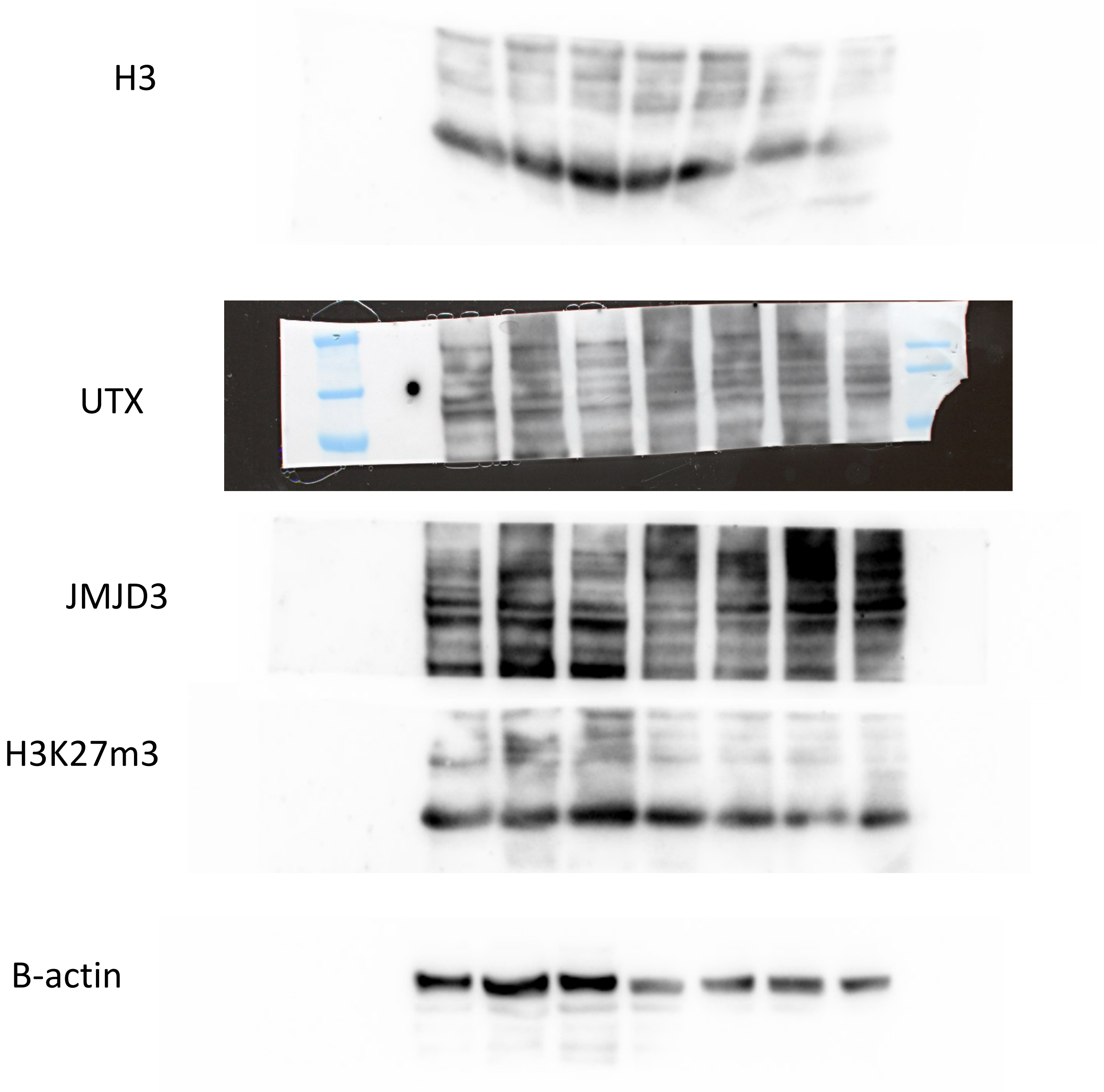

Western Blot (Supplementary Figure S4B)

Cal33, n2

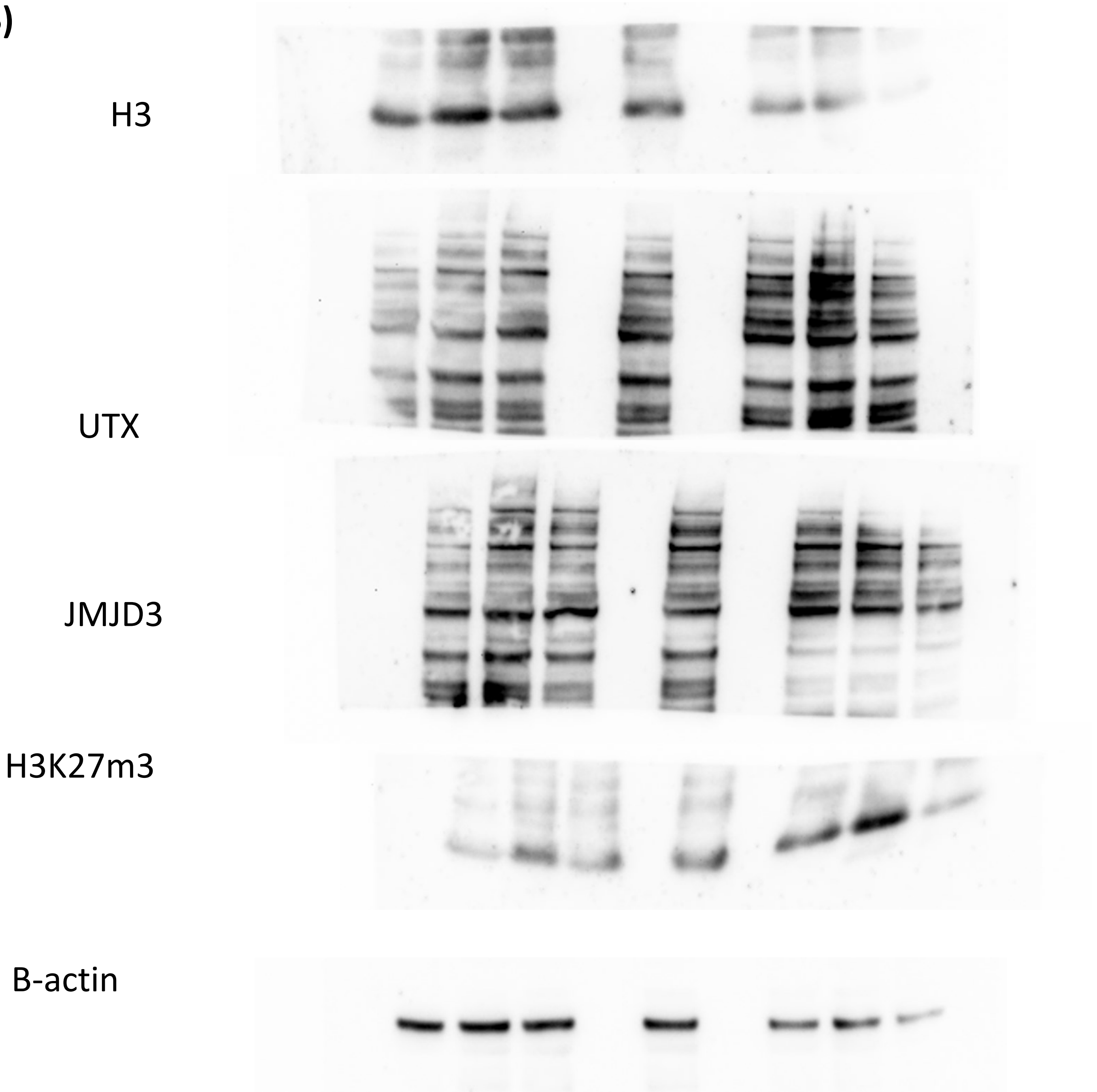

Western Blot (Supplementary Figure S4B)

Cal33, n3

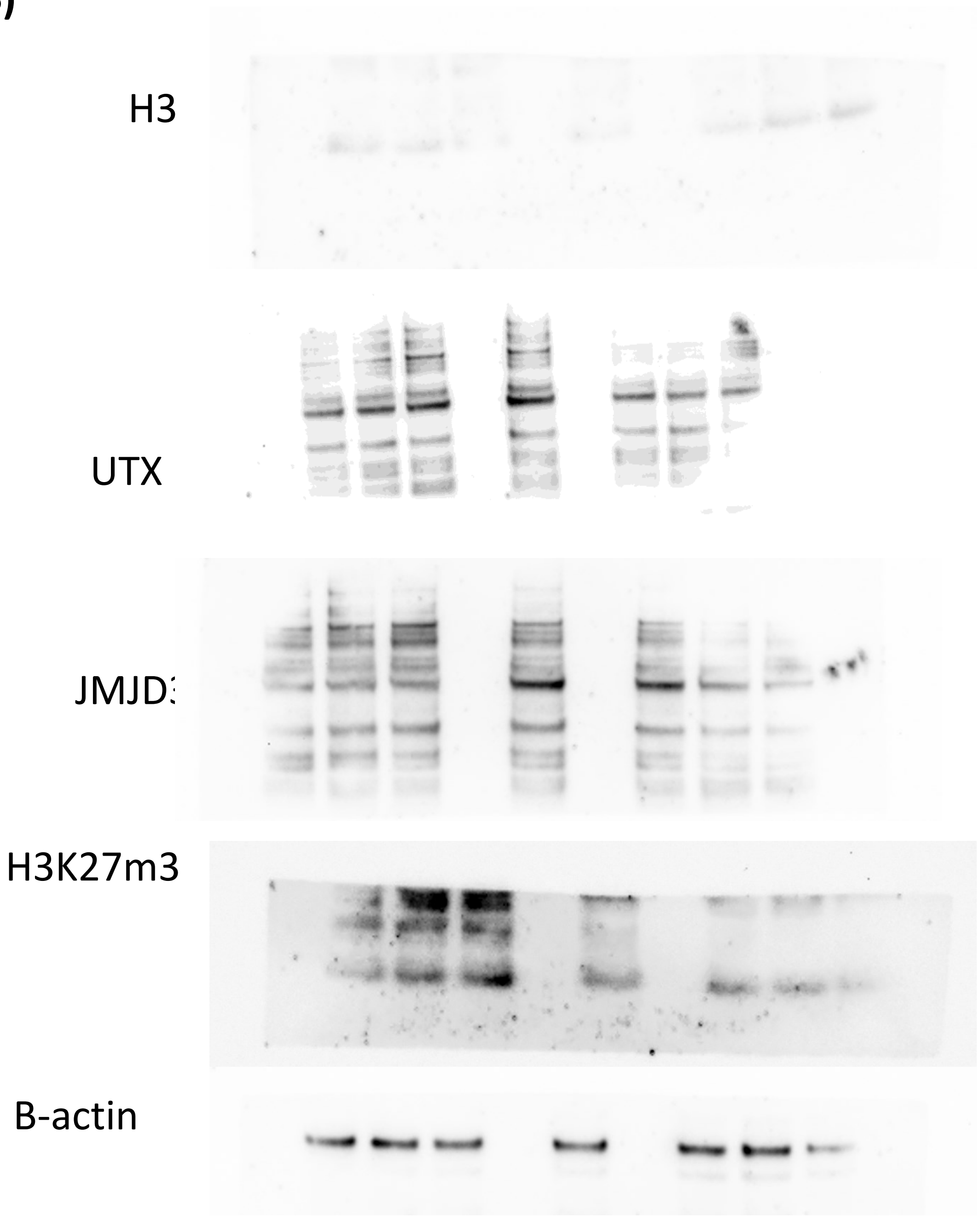

KDM6A-Composite

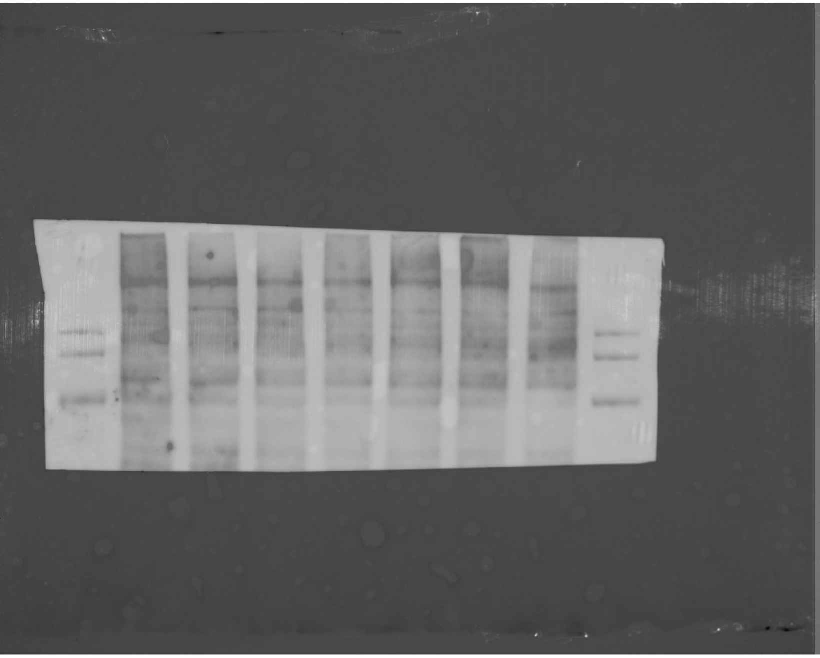

KDM6B-Composite

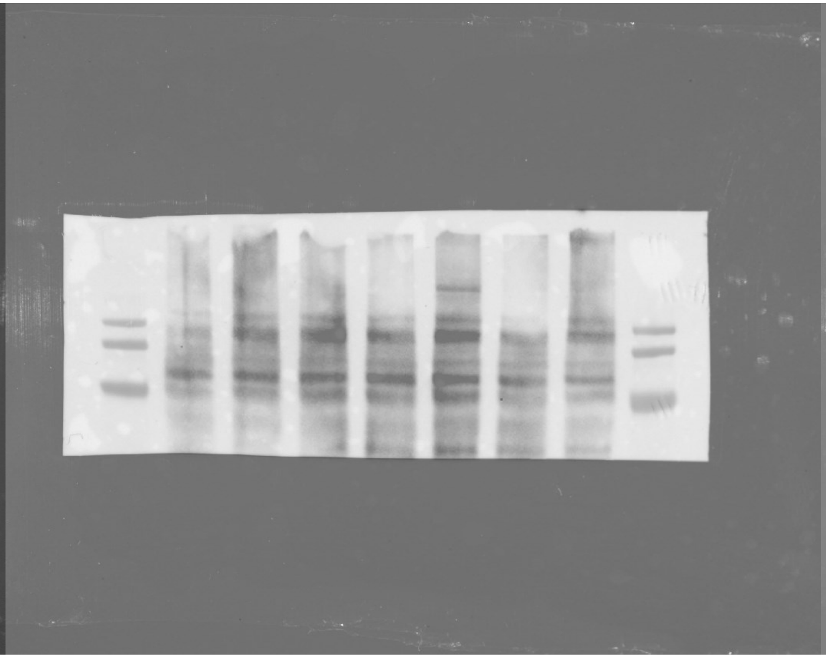

H3-Composite

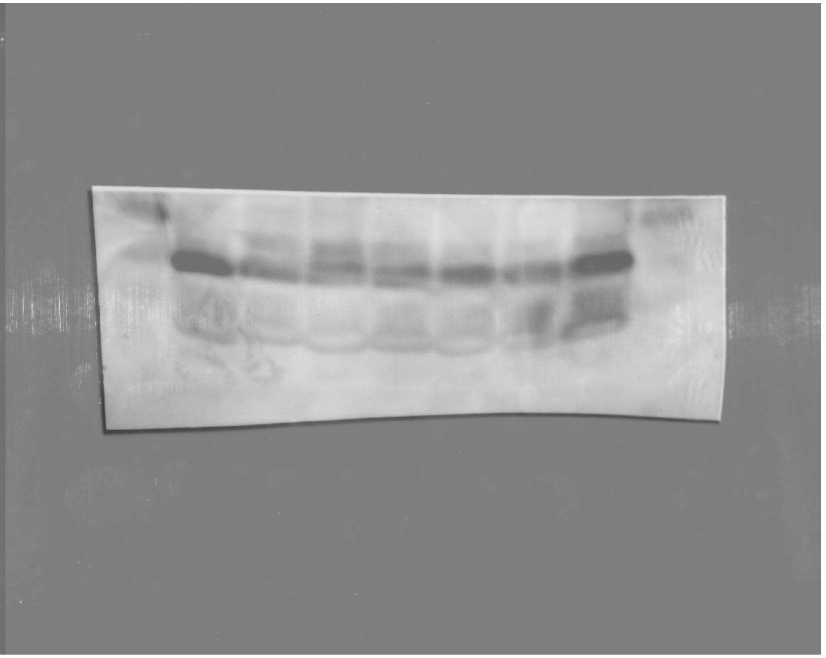

KDM6A-Chemiluminescence

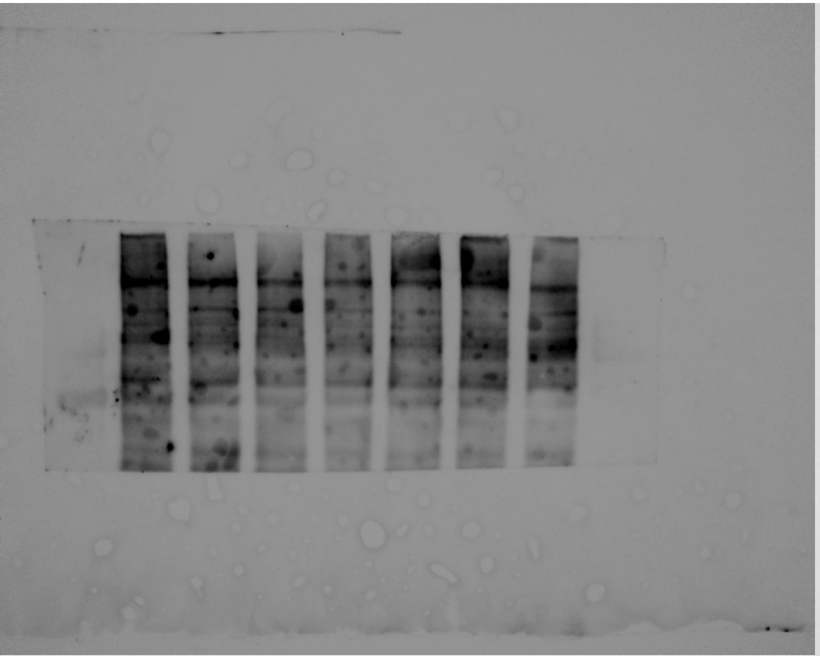

KDM6B-Chemiluminescence

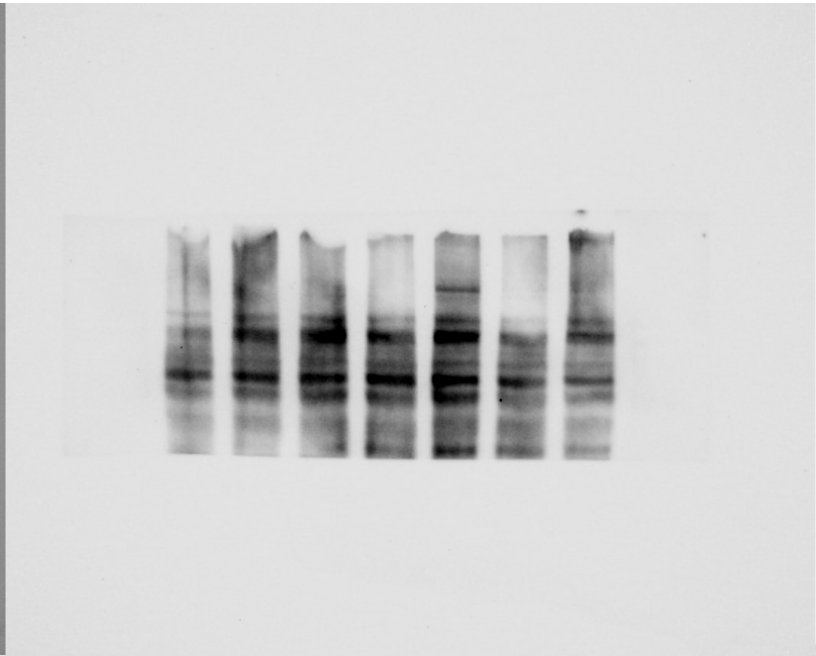

H3-Chemiluminescence

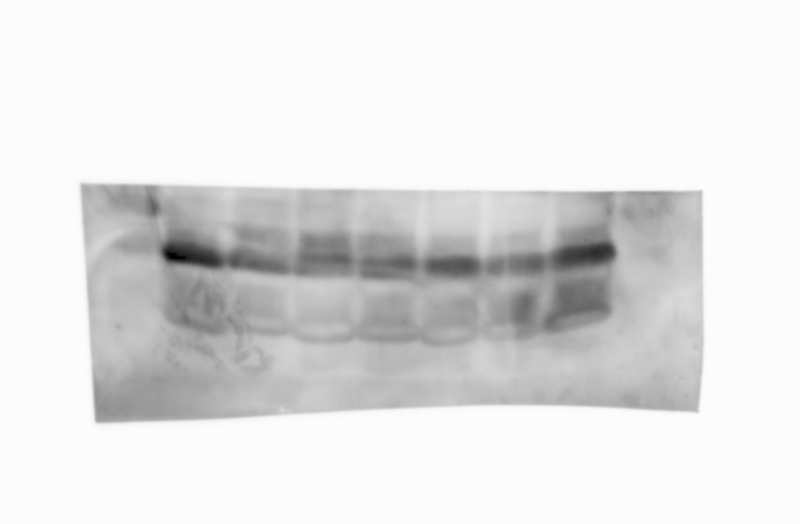

Western Blot (Supplementary Figure S4B)

SAS, n2

B-Actin-Membran2-Composite

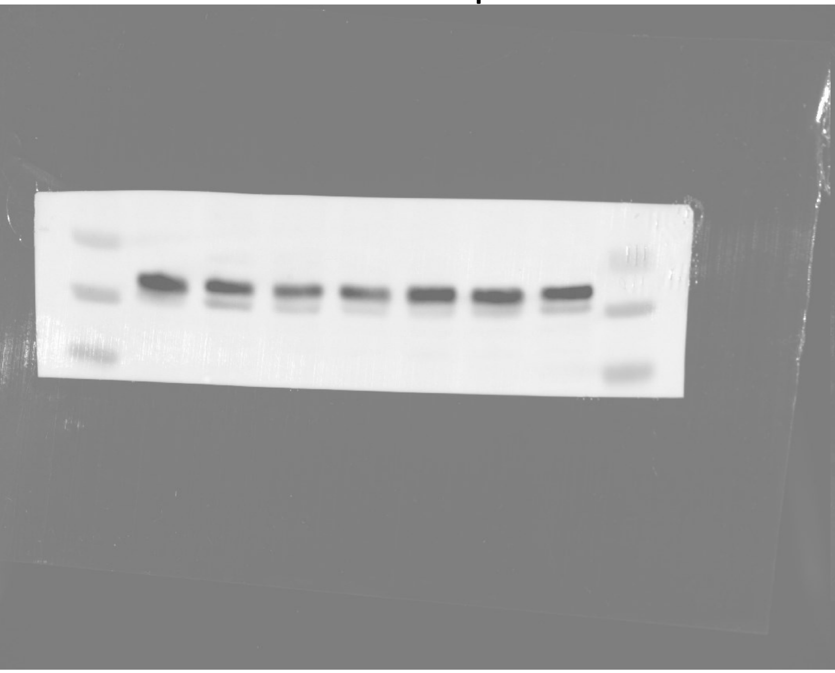

B-Actin-Membran1-Composite

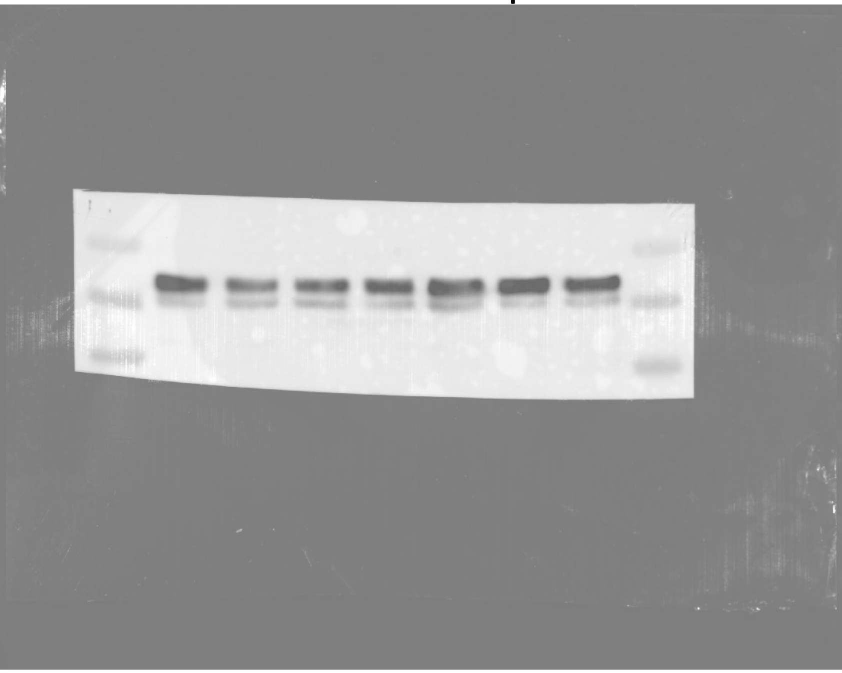

H3K27me3-Composite

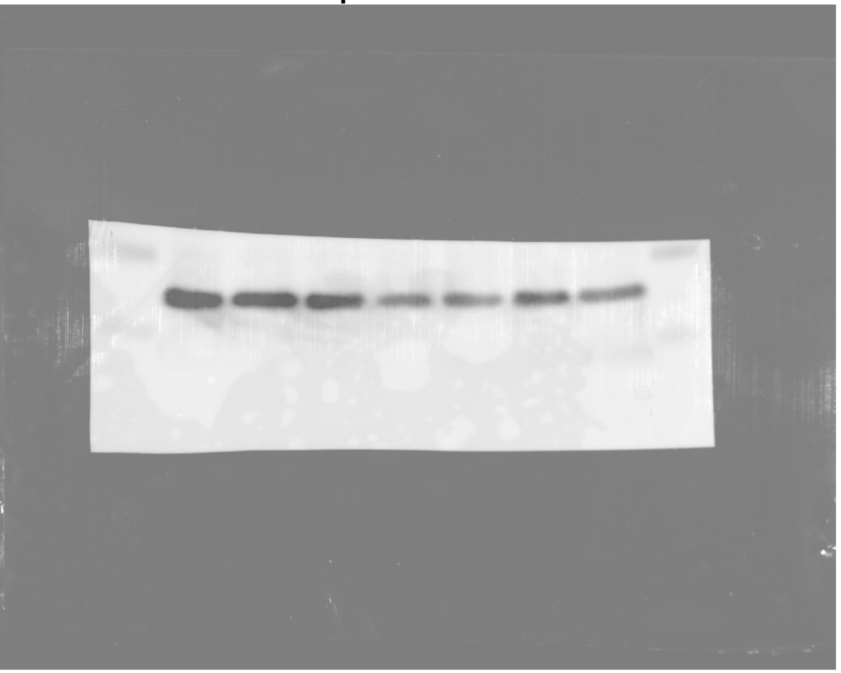

B-Actin-Membran2-Chemiluminescence

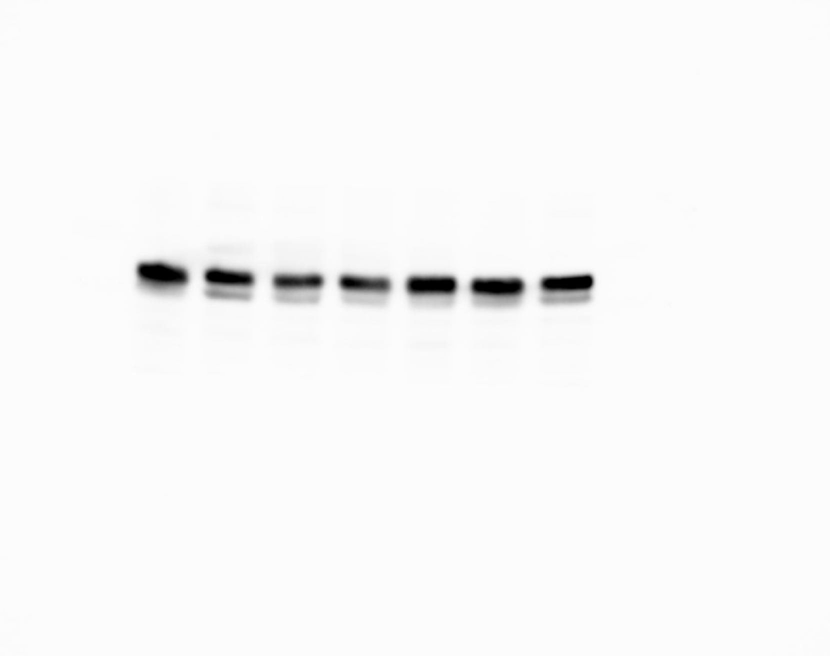

B-Actin-Membran1-Chemiluminescence

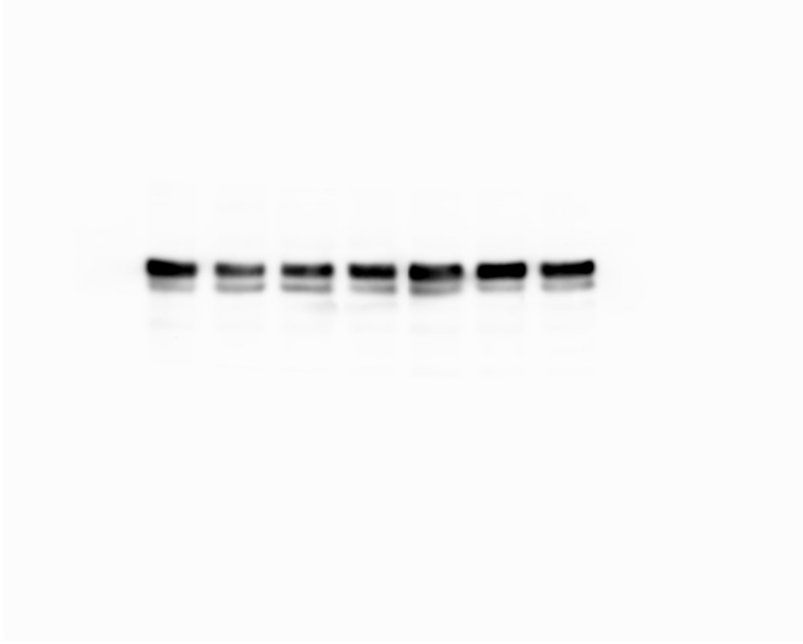

H3K27me3-Chemiluminescence

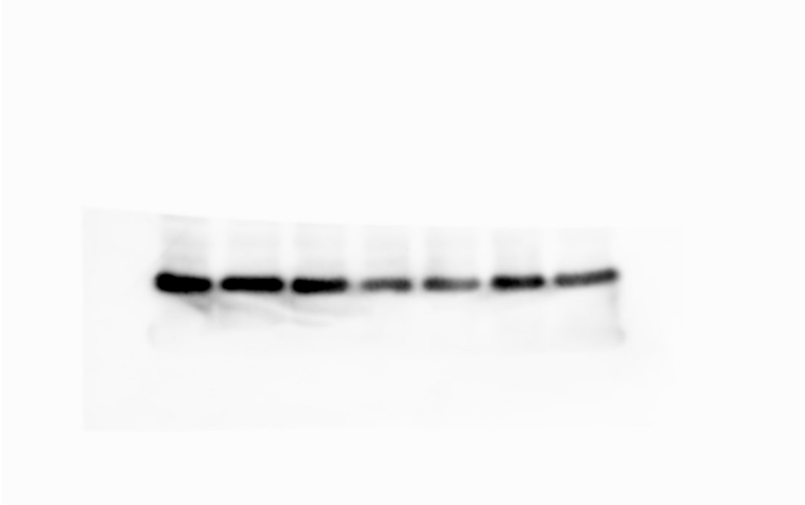

Western Blot (Supplementary Figure S4B)

SAS, n2

Western Blot (Supplementary Figure S4B)

SAS, n4

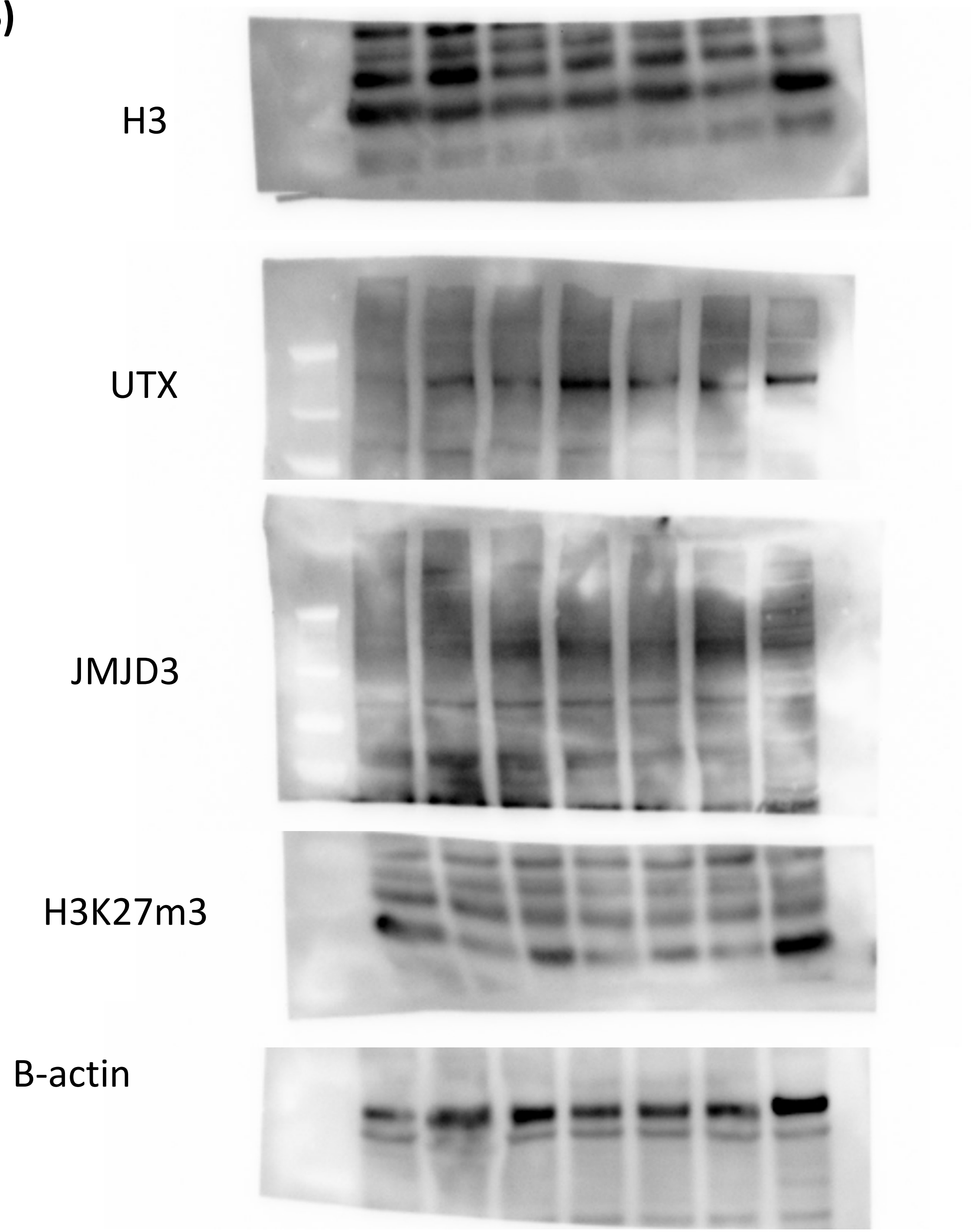

Western Blot (Supplementary Figure S1A)

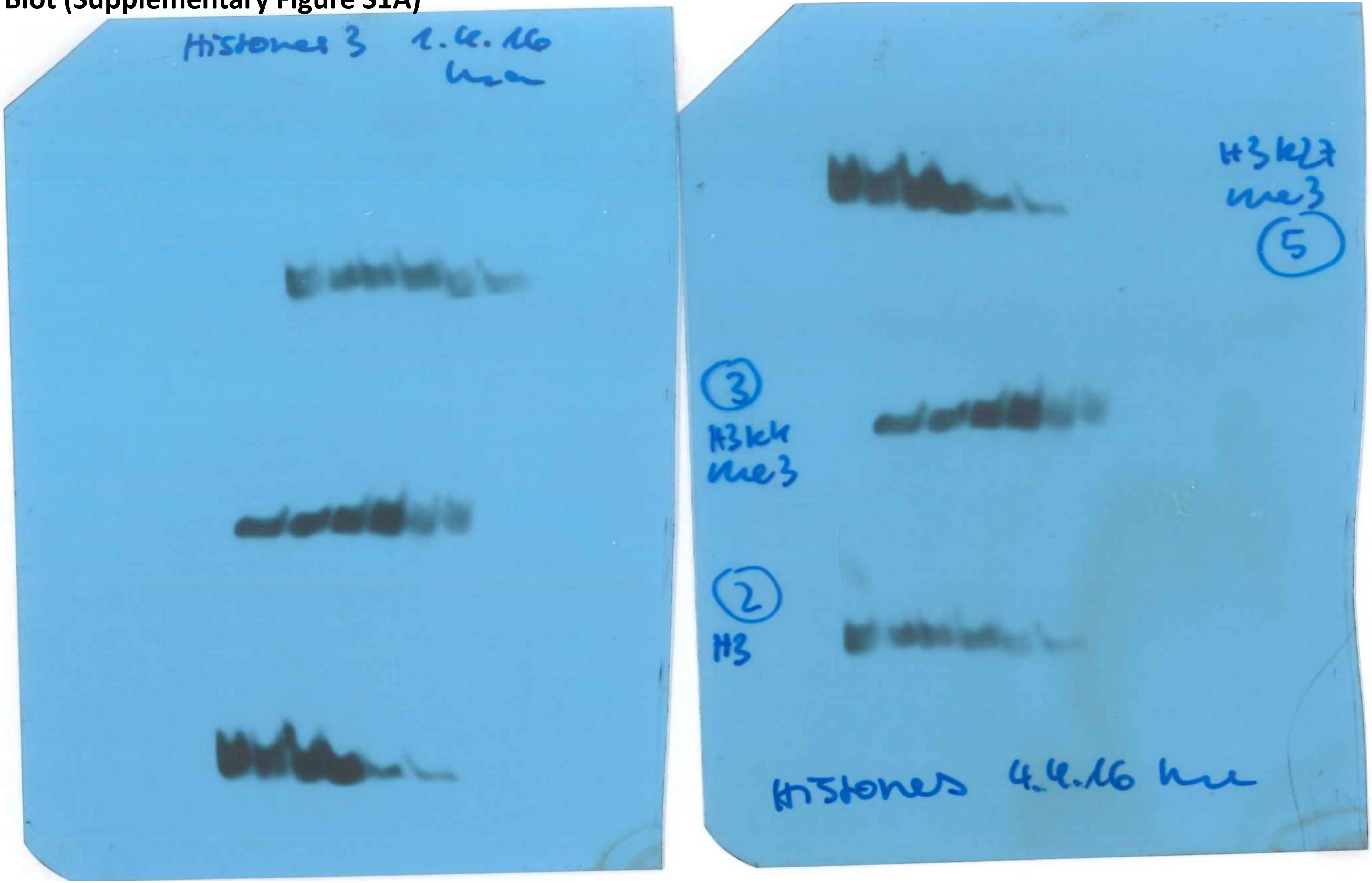

Western Blot (Supplementary Figure S1A)

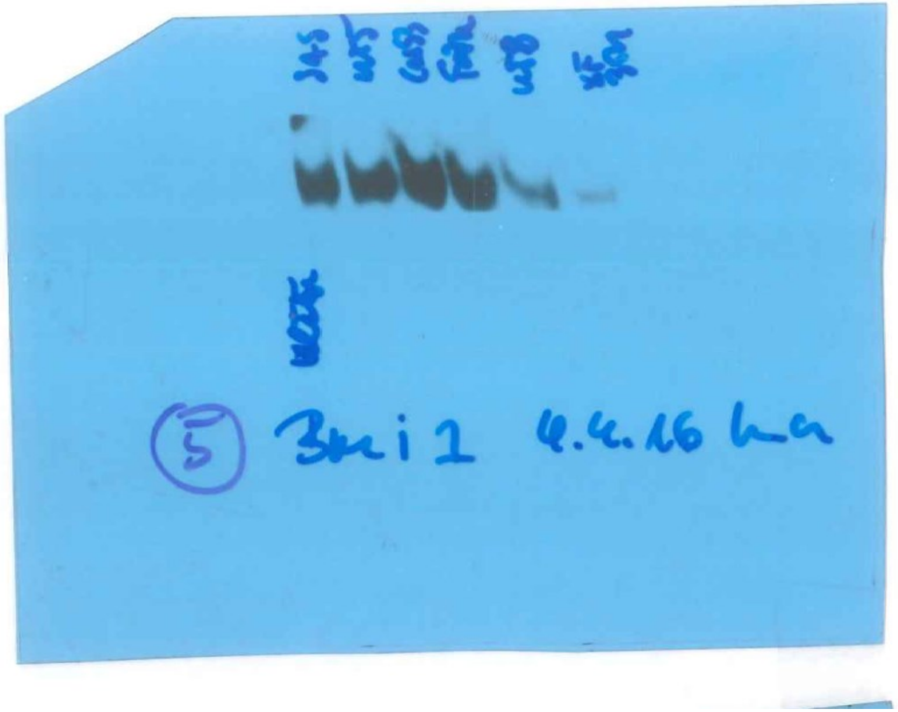

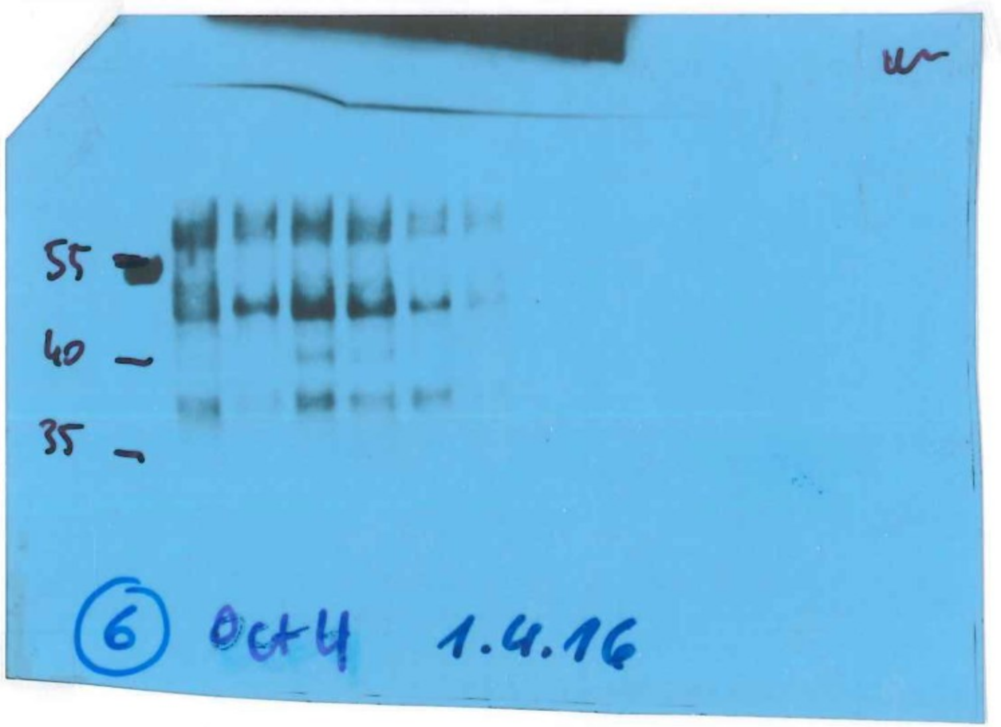

Western Blot (Supplementary Figure S1A)

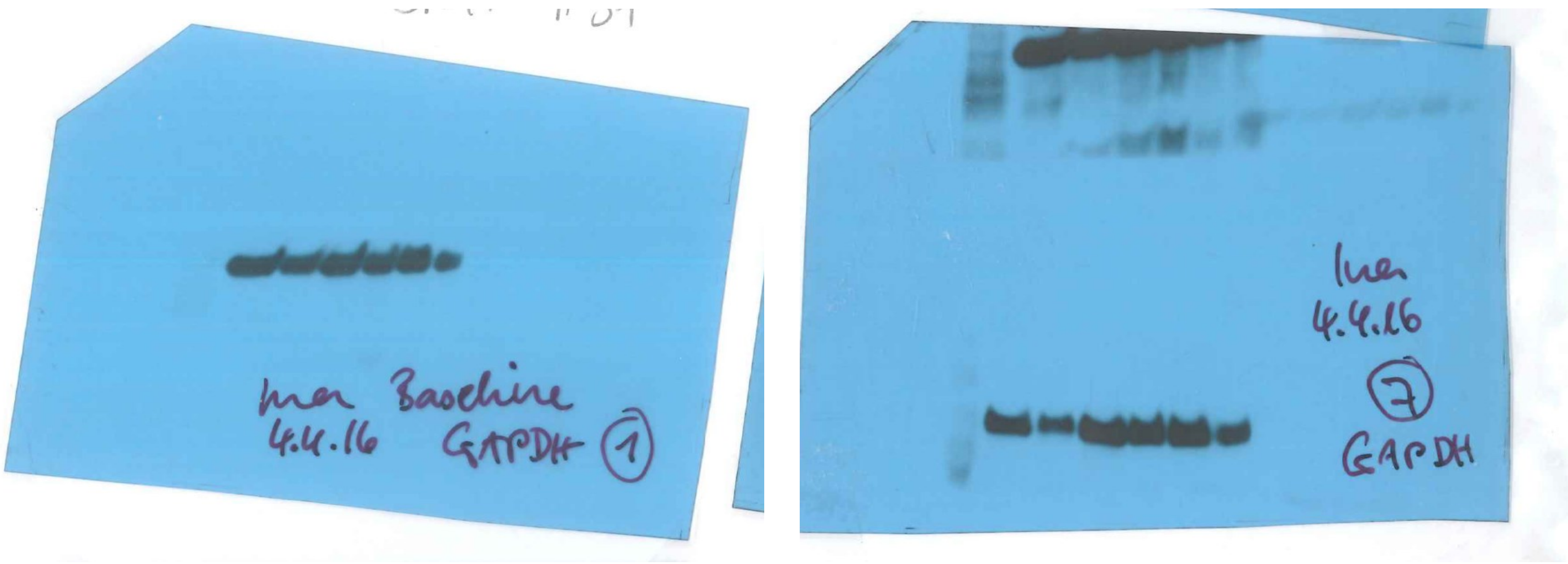

Supplement: Supplementary file 1 [file cancers-16-00730-s001.zip › Raw western for Fis. 5C, S1A and S4B.pdf]
